# Variations in the quality of tuberculosis care in urban India: A cross-sectional, standardized patient study in two cities

**DOI:** 10.1371/journal.pmed.1002653

**Published:** 2018-09-25

**Authors:** Ada Kwan, Benjamin Daniels, Vaibhav Saria, Srinath Satyanarayana, Ramnath Subbaraman, Andrew McDowell, Sofi Bergkvist, Ranendra K. Das, Veena Das, Jishnu Das, Madhukar Pai

**Affiliations:** 1 Development Research Group, The World Bank, Washington, District of Columbia, United States of America; 2 University of California at Berkeley, Berkeley, California, United States of America; 3 Institute for Socio-Economic Research on Development and Democracy, Delhi, India; 4 Center for Operational Research, International Union Against TB and Lung Diseases, Paris, France; 5 Department of Public Health and Community Medicine, Tufts University School of Medicine, Boston, Massachusetts, United States of America; 6 Centre National de la Recherche Scientifique, Paris, France; 7 ACCESS Health International, New York, New York, United States of America; 8 Department of Anthropology, Johns Hopkins University, Baltimore, Maryland, United States of America; 9 Center for Policy Research, New Delhi, India; 10 McGill International TB Centre, McGill University, Montreal, Canada; 11 Manipal McGill Centre for Infectious Diseases, Manipal Academy of Higher Education, Manipal, India; Universidad Peruana Cayetano Heredia, PERU

## Abstract

**Background:**

India has the highest burden of tuberculosis (TB). Although most patients with TB in India seek care from the private sector, there is limited evidence on quality of TB care or its correlates. Following our validation study on the standardized patient (SP) method for TB, we utilized SPs to examine quality of adult TB care among health providers with different qualifications in 2 Indian cities.

**Methods and findings:**

During 2014–2017, pilot programs engaged the private health sector to improve TB management in Mumbai and Patna. Nested within these projects, to obtain representative, baseline measures of quality of TB care at the city level, we recruited 24 adults to be SPs. They were trained to portray 4 TB “case scenarios” representing various stages of disease and diagnostic progression. Between November 2014 and August 2015, the SPs visited representatively sampled private providers stratified by qualification: (1) allopathic providers with Bachelor of Medicine, Bachelor of Surgery (MBBS) degrees or higher and (2) non-MBBS providers with alternative medicine, minimal, or no qualifications.

Our main outcome was case-specific correct management benchmarked against the Standards for TB Care in India (STCI). Using ANOVA, we assessed variation in correct management and quality outcomes across (a) cities, (b) qualifications, and (c) case scenarios. Additionally, 2 micro-experiments identified sources of variation: first, quality in the presence of diagnostic test results certainty and second, provider consistency for different patients presenting the same case.

A total of 2,652 SP–provider interactions across 1,203 health facilities were analyzed. Based on our sampling strategy and after removing 50 micro-experiment interactions, 2,602 interactions were weighted for city-representative interpretation. After weighting, the 473 Patna providers receiving SPs represent 3,179 eligible providers in Patna; in Mumbai, the 730 providers represent 7,115 eligible providers. Correct management was observed in 959 out of 2,602 interactions (37%; 35% weighted; 95% CI 32%–37%), primarily from referrals and ordering chest X-rays (CXRs). Unnecessary medicines were given to nearly all SPs, and antibiotic use was common. Anti-TB drugs were prescribed in 118 interactions (4.5%; 5% weighted), of which 45 were given in the case in which such treatment is considered correct management.

MBBS and more qualified providers had higher odds of correctly managing cases than non-MBBS providers (odds ratio [OR] 2.80; 95% CI 2.05–3.82; *p* < 0.0001). Mumbai non-MBBS providers had higher odds of correct management than non-MBBS in Patna (OR 1.79; 95% CI 1.06–3.03), and MBBS providers’ quality of care did not vary between cities (OR 1.15; 95% CI 0.79–1.68; *p* = 0.4642). In the micro-experiments, improving diagnostic certainty had a positive effect on correct management but not across all quality dimensions. Also, providers delivered idiosyncratically consistent care, repeating all observed actions, including mistakes, approximately 75% of the time. The SP method has limitations: it cannot account for patient mix or care-management practices reflecting more than one patient–provider interaction.

**Conclusions:**

Quality of TB care is suboptimal and variable in urban India’s private health sector. Addressing this is critical for India’s plans to end TB by 2025. For the first time, we have rich measures on representative levels of care quality from 2 cities, which can inform private-sector TB interventions and quality-improvement efforts.

## Abstract 2

Madhukar Pai and colleagues use standardized patients trained to portray symptoms of tuberculosis to assess the quality of diagnosis and care across private sector health providers in Mumbai and Patna, India.

## Author summary

**Why was this study done?:**

- India accounts for a quarter of the world’s estimated 10.4 million new tuberculosis (TB) cases per year, nearly a third of the 1.7 million annual TB deaths, and a third of the estimated 4 million “missing patients” who are either not diagnosed or are not reported to national TB programs.
- The private health sector provides the bulk of primary care in India, serving as the first point of contact for 50% to 70% of patients with TB symptoms. The private sector comprises a wide range of qualification levels: allopathic providers with Bachelor of Medicine, Bachelor of Surgery (MBBS) or higher degrees; Ayurveda, Yoga, Unani, Siddha, or Homeopathy (AYUSH) practitioners (practitioners with degrees in alternative medicine or traditional systems of health); and providers with other or no formal qualifications, who are known to provide a large proportion of primary care.
- Little is known about quality of TB care in the private sector; however, the Government of India has prioritized the engagement of the private sector in the National Strategic Plan (NSP) for TB elimination (2017–2025).

**What did the researchers do and find?:**

- In the 2 Indian cities of Mumbai and Patna, we used a list of all private-sector providers to construct a representative sample for a quality of care surveillance project using the standardized patient (SP) method.
- During 2014–2015, the SPs—seemingly healthy adults hired locally and trained to convincingly portray symptoms of illness or test results to providers—conducted a total of 2,652 incognito visits among 1,203 health facilities. SPs portrayed cases representing 4 different stages of TB progression and diagnosis and reported the providers’ actions and prescriptions for analysis.
- We report 4 novel findings in this publication. First, benchmarked against national and international standards of TB care, only 35% of interactions resulted in standards-compliant care during a one-time visit to a healthcare provider.
- Second, lack of adherence to TB standards does not represent a typical “alternative” care pattern that would be appropriate for polluted urban environments because providers followed multiple different protocols.
- Third, there is a wide range of estimated quality in each qualification stratum, and providers demonstrate consistency with their own previous behaviors.
- Finally, providers offer more TB-focused care in cases with higher diagnostic certainty provided by the patient.

**What do these findings mean?:**

- Although the SP method deployed in this study cannot account for patient mix or care-management practices reflecting more than one patient–provider interaction, we find that quality of TB care is suboptimal and variable in urban India’s private health sector. Therefore, improving quality of TB management in the private sector must be a priority for India’s TB elimination strategy.
- With large-scale, city-level representative estimates, these findings can inform specific efforts for private-sector TB interventions and quality improvement.

## Introduction

India accounts for a quarter of the estimated 10.4 million new tuberculosis (TB) cases worldwide annually, nearly a third of the 1.7 million annual TB deaths, and a third of the estimated 4 million “missing patients” who are either not diagnosed or are not reported to national TB programs [1]. Identifying these missing patients with TB, accurately diagnosing patients in a timely manner, and providing all patients with quality treatment is critical for reducing TB incidence and mortality rates [2].

In India, studies have demonstrated that the private health sector provides the bulk of primary care, is the first point of contact for 50% to 70% of patients with TB symptoms [3–5], and prescribes nearly twice the amount of anti-TB drugs compared to the public sector [6]. However, navigating the private health sector can be medically and economically costly: patients with TB who seek care experience a median of 33.5 days of diagnostic and treatment delays, convoluted pathways with multiple visits to an average of 3 providers before diagnosis, and a broken cascade of care even if diagnosed [4,5,7,8]. With the emergence of drug-resistant strains, these deficits may perpetuate disease transmission and hinder control efforts, particularly for high-density urban areas [9].

The Government of India now recognizes that engaging the private sector is critical to TB control, and it has included this as an explicit goal in the government’s National Strategic Plan (NSP) for TB elimination (2017–2025). The NSP articulates a commitment to massively expand private provider engagement and calls for a 6-fold increase in TB case notifications from the private sector, to 2 million patients per year by 2020 [10].

One key challenge for any effort that engages the private sector is the wide array of healthcare providers, most of whom are not engaged with India’s Revised National TB Control Programme (RNTCP) [11]. The sector comprises providers trained in biomedicine with Bachelor of Medicine, Bachelor of Surgery (MBBS) or higher degrees (such as MDs and specialized MDs), practitioners of alternative medicine and healthcare (such as those who hold degrees in Ayurveda, Yoga, Unani, Siddha, or Homeopathy [abbreviated AYUSH]), informal providers with minimal or no qualifications, and pharmacists and laboratories.

All private providers with MBBS or higher qualifications can provide TB care, and AYUSH practitioners, other non-MBBS providers, and pharmacists generally do not treat TB [12–14]. TB medications are included in Schedule H1, a restricted drug list, in India and therefore can only be dispensed on the basis of prescriptions from MBBS or higher providers. Additional evidence demonstrates that pharmacists do not dispense TB medications over the counter [13,14].

In the private sector, there is no uniform diagnostic algorithm that is practiced, and research demonstrates that providers use diverse approaches (e.g., clinical diagnosis only and clinical diagnosis with chest X-ray [CXR] confirmation, with infrequent use of microbiological tests) [12,15]. Xpert Mycobacterium tuberculosis/Rifampicin (Xpert MTB/RIF), also known as GeneXpert (Cepheid, Sunnyvale, CA), a molecular diagnostic test that detects TB as well as rifampicin-resistant TB, is available in certain private laboratories, and anecdotal evidence suggests that providers use these diagnostics selectively based on patients’ ability to pay and patient risk factors, such as suspected drug resistance. Effective private-sector engagement therefore demands a deep understanding of how private providers behave and what predicts their practice patterns [11].

Between 2014 and 2016, the Bill & Melinda Gates Foundation India Country Office, along with city and state governmental partners, funded 2 Private Provider Interface Agency (PPIA) pilot programs in Mumbai and Patna. The PPIA pilots were private-sector engagement efforts to increase case notifications and improve TB management. Our study was a part of this larger effort to improve quality of care in both cities. Using the baseline data from the quality of care surveillance, the objectives of this study are to answer the following 2 research questions: (1) what are city-representative levels of quality of TB care in urban India across private provider qualifications and cities and (2) to what extent can variation in quality of care be explained?

In this study, we build on our extensive prior work that validated the use of the standardized (or simulated) patient (SP) method for assessing TB care quality and for directly measuring levels of quality of TB care in India, China, and Kenya [12,14,16–18]. The SP method, which is considered the gold standard method to assess provider practice, has been increasingly used to capture levels of quality of care for TB and other health conditions [12,16–20]. In order to provide city-representative measures on what explains levels of quality, we report data on 2,602 SP–provider interactions across 1,203 representatively sampled health facilities in Mumbai and Patna. We additionally report results from 2 micro-experiments aimed to explain variation in our main outcome of interest: correct case management during a single encounter with a provider.

## Methods

### Study settings

We conducted this study in Mumbai and urban Patna, when the 2 PPIA pilots were in the first year of implementation [21,22]. Patna is the capital of the state of Bihar, one of the least developed Indian states, with an annual per capita income of 30,000 Indian rupees (INR; US$470), and urban Patna has a population of 2.049 million city inhabitants (2011 census figure). Mumbai is the relatively wealthier port capital of the state of Maharashtra and is home to 12 million inhabitants (2011 census figure), with an annual per capita income of 180,000 INR (US$2,845).

Although both cities have government clinics and hospitals for patients, the mostly unregulated private sector remains the dominant source of primary healthcare. However, the structure of the private sector is very different across these 2 cities. In Patna, MBBS-qualified providers tend to operate stand-alone, single-provider clinics, whereas in Mumbai, they work in several stand-alone, single-provider clinics as well as multiple multiprovider facilities or in hospitals with a mix of MBBS providers and specialists with higher qualifications. Patna non-MBBS providers tend to be those with other or no qualifications, whereas in Mumbai, non-MBBS providers are predominantly AYUSH practitioners. In this paper, we refer to the group of allopathic providers with MBBS degrees or higher qualifications as “MBBS providers” and to all others as “non-MBBS providers” regardless of the specific qualifications of the providers, in accordance with our sampling stratification.

In the TB context, 97,001 patients with TB were officially notified in 2016 in the state of Bihar, and 195,139 cases were notified in the state of Maharashtra [23]. Because, as studies suggest, most TB cases are treated in the private sector, many other cases are not notified to national TB authorities, and therefore the above numbers underestimate the extent of the disease burden. The prevalence of drug-resistant TB strains is increasing in both cities, and drug-resistance concerns have been prominent in Mumbai, particularly with the emergence of totally drug-resistant TB strains [24].

### Study design

The SP survey methodology consists of 3 steps: creating a sampling frame, measuring quality, and analyzing the resulting data. We briefly describe each step here, with additional details in Supporting information (S1–S3 Text).

#### Provider and facility sampling

In both Mumbai and Patna, field officers hired by the PPIAs conducted street-by-street mapping exercises in 2014 to construct a comprehensive, “universe” list of all providers and facilities across the private health sectors of both cities. Providers eligible for the SP study were restricted to those known to see adult outpatients with respiratory symptoms in the private health sector. These include most primary care providers but exclude, for instance, orthopedists, gynecologists, ophthalmologists, and pediatricians. Using the universe list, we then representatively sampled eligible providers from purposively sampled geographical areas within each city, which were identified with implementation partners for the SP study in the context of the PPIA pilot programs. Providers were selected with random sampling stratified by PPIA program enrollment status and provider qualification (Table 1). This was done to estimate baseline measures for quality of TB care in these cities and, in ongoing work, evaluate the impact of the programs (see S1 Text) (we note that a stratified analysis by PPIA versus non-PPIA is not a focus of this paper but a focus of the larger impact evaluation study that is ongoing). In Mumbai, we sampled 331 MBBS facilities and 500 non-MBBS providers, and in Patna, we sampled 471 MBBS and 120 non-MBBS providers (S7 Fig). To estimate baseline quality measures, the data were weighted according to appropriate sampling proportions in both cities (S2 Text).

**Table 1:** Sampling and weighting descriptions.

Sample Description	Number in City	Sampling Methodology	Number in Data	Case Assignment	Case 1 Interactions	Case 1 Weight	Case 2 Interactions	Case 2 Weight	Case 3 Interactions	Case 3 Weight	Case 4 Interactions	Case 4 Weight
**Patna: non-MBBS, non-PPIA**	**1,074**	Random sample drawn from Patna block 34/73 wards, Danapur block 40/40 wards, Phulwari Sharif block 28/28 wards and stratified by qualification and PPIA status as of September 25, 2014.	59	Each provider was assigned 2 cases. Case 1 was assigned to all providers. Then, each provider was randomly assigned to also receive Case 2, Case 3, or Case 4 (1:1:1 ratio). Non-MBBS providers who had already received Case 1 by December 31, 2014 were selected to receive an identical Case 1 (“repeat Case 1 visit”) portrayed by a different SP between January 8, 2015 and February 17, 2015.	91	0.00371	20	0.01689	20	0.01689	18	0.01877
**Patna: non-MBBS, PPIA**	**264**		60		93	0.00089	20	0.00415	20	0.00415	20	0.00415
**Patna: MBBS, non-PPIA**	**1,642**	Random sample drawn from Patna block 34/73 wards, Danapur block 40/40 wards, Phulwari Sharif block 28/28 wards and stratified by qualification and PPIA status as of January 2015.	256	Each provider was assigned 2 cases. Case 1 was assigned to all providers. Then, each provider was randomly assigned to also receive Case 2, Case 3, or Case 4 (1:1:1 ratio). MBBS providers who had already received Case 1 by December 31, 2014 were selected to receive an identical Case 1 (“repeat Case 1 visit”) portrayed by a different SP between January 8, 2015 and February 17, 2015.	253	0.00204	70	0.00738	77	0.00671	85	0.00608
**Patna: MBBS, PPIA**	**199**		98		136	0.00046	28	0.00224	33	0.00190	35	0.00179
**Mumbai: non-MBBS, non-PPIA**	**3,330**	Drawn from 4 purposively selected high–TB-burden and high–slum-population wards and stratified by qualification and PPIA status as of January 24, 2014.	418	Each provider was assigned 2 cases. Each provider was assigned Case 1. Then, each provider was randomly assigned to receive Case 2, Case 3, or Case 4 (1:1:2 ratio).	412	0.00114	104	0.00450	103	0.00454	205	0.00228
**Mumbai: non-MBBS, PPIA**	**261**		87		87	0.00042	21	0.00175	22	0.00167	42	0.00087
**Mumbai: MBBS, non-PPIA**	**3,374**	Facility-level: PPIA hospital (i.e., “hub”) census from entire Mumbai and comparable and purposively selected non-PPIA from 4 wards (same as non-MBBS sample wards) with PPIA status as of April 2015. Provider-level: Provider selection to maximize sample size drawn from PPIA hospitals only from 18 high–TB-burden and high–slum-population wards and based on PPIA activity as of April 2015.	127	PPIA hubs were assigned 1 SP walk-in, and non-PPIA hubs were assigned 2 or 3 SP walk-ins. PPIA hubs received SP1 only for walk-ins. Non-PPIA hubs were all assigned SP1 walk-ins; a random half were assigned SP2; a random half were assigned SP3, with the other half assigned SP4 without a sputum report. Providers at networked locations were assigned 2–4 SPs given existing knowledge at the time of scheduling. All providers received SP1, but PPIA providers who saw an SP1 during walk-ins were not assigned another SP1. A random half were assigned SP2. A random half were assigned SP3 with a sputum report and SP4 without a sputum report; the other half received SP4 with a sputum report (the experimental subsample).	134	0.00354	69	0.00687	28	0.01693	30	0.01580
**Mumbai: MBBS, PPIA**	**150**		98		171	0.00012	53	0.00040	51	0.00042	51	0.00042

**Abbreviations:** MBBS, Bachelor of Medicine, Bachelor of Surgery; PPIA, Private Provider Interface Agency; SP, standardized patient; TB, tuberculosis.

SP cases were then assigned to providers to ensure that (a) providers of all types would receive a mix of multiple SP case scenarios and (b) the risk of detection would be minimized (i.e., in order to minimize the likelihood of providers being suspicious of SPs or detecting SPs as not real patients, the schedule for SP visits were conducted such that providers would not receive more than 2 SP cases within 5 days of each other and that Case 1 would visit before Cases 2–4 in order to prevent any potential priming effect from a provider seeing a more advanced or obvious TB case before a less advanced one).

Interaction completion rates were uniformly high, with only Patna MBBS, non-PPIA providers having less than 85% of initially scheduled providers successfully visited (this proportion was 71%, due to many being discovered as specialized practitioners who did not treat respiratory conditions). When interactions could not be completed at an initially scheduled provider, they were completed at an identically sampled replacement if possible (S1 Text); therefore, the total number of unique providers in the data is greater than the number initially sampled. Each provider in our sample received up to 4 SP visits at a given location.

#### Measuring quality using SPs

The advantages of SP-based measures of medical care quality in general [16,25] and for TB specifically have been discussed previously [12,14]. For this study, 4 distinct SP cases were developed and agreed upon by a Technical Advisory Group (TAG) made up of clinicians, economists, anthropologists, experts in international and national TB guidelines, and other stakeholders (S1 Text), after which the cases were validated in a pilot study [12]. Our main outcome, correct management, was benchmarked against the Standards for TB Care in India (STCI) and the International Standards for TB Care (ISTC) and was agreed upon by the TAG [26,27] (Table 2). Each case was developed with a standardized opening statement and scripted presentation that would advance the provider towards a TB diagnosis and an appropriate case management action, which could include referral, laboratory testing, or treatment initiation, depending on the case scenario.

**Table 2:** SP case descriptions, patient presentations, and correct management definitions.

SP Case	Case Description	Presentation of Patient	Expected Correct Case Management
**Case 1: Naïve Suspected TB**	Classic case of presumed TB with 2–3 weeks of cough and fever.	Presents with presumptive TB, for the first time, to a private healthcare provider, saying “Doctor, I have a cough that is not getting better and some fever too.”	Recommendation for sputum testing, chest radiograph, or referral to a public DOTS center or a private provider or specialist
**Case 2: Suspected TB with Abnormal CXR**	Classic case of presumed TB in a patient who has had 2–3 weeks of cough and fever. The patient has taken a broad-spectrum antibiotic (amoxicillin) given by another healthcare provider for 1 week with no improvement. He also carries an abnormal CXR suggestive of TB.	Presents after an initial, failed (empirical) treatment for symptoms with broad-spectrum antibiotics and a diagnostic CXR, saying “I have a cough and fever which is not getting better. I went to a doctor and took the medicines he gave me and have also had an X-ray done.” The CXR and blister pack for the antibiotics are shown if the provider asks.	Recommendation for sputum testing, chest radiograph, or referral to a public DOTS center or a private provider or specialist
**Case 3: TB Case**	Chronic cough with a positive sputum smear report for TB from a public health facility.	Presents with evidence of microbiologically confirmed TB, saying “I have had a cough for nearly a month now and also have fever. I visited [the local government hospital] and they gave me some medicines and did a sputum test.” The sputum report is shown if the provider asks.	Either referral to a public DOTS center, a private provider or specialist, or (in the case of a qualified private provider) initiation of treatment with standard, 4-drug, first-line anti-TB therapy (HRZE regimen)
**Case 4: Suspected MDR**	Chronic cough and, if asked, elaborates a history of previous, incomplete treatment for TB, which would raise the suspicion of MDR TB.	Presents as a previously treated patient with TB with recurrence of the disease (i.e., suspicion of drug resistance), saying “Doctor, I am suffering from a bad cough. One year ago, I got treatment in [the local public hospital], and it had gotten better. But now I am having cough again.”	Recommendation for any DST (culture, line probe assay, or Xpert MTB/RIF) or referral to a public DOTS center or to a private provider or specialist

**Abbreviations:** CXR, chest X-ray; DOTS, directly observed treatment, short form; DST, drug susceptibility test; HRZE, isoniazid, rifampicin, pyrazinamide, and ethambutol; MDR, multidrug resistant; SP, standardized patient; TB, tuberculosis; Xpert MTB/RIF, Xpert Mycobacterium tuberculosis/Rifampicin.

Cases 1, 2, and 3 represented adult pulmonary TB at various stages of diagnostic certitude. Case 1 presents as a classic case of pulmonary TB with 2 to 3 weeks of cough and fever. Case 2 is similar to Case 1; however, the SP additionally has completed a 1-week course of broad-spectrum antibiotics without any improvement and carries an abnormal CXR dated within 2 weeks of the interaction. Case 3 has visited the local government hospital and carries the results of a sputum smear microscopy acid-fast bacillus (AFB) test, which is positive for active TB.

Similar to previously published SP studies, the SPs in this study were recruited from the local community, hired, and extensively trained to present the same case to multiple providers. Interaction details were recorded by field supervisors in a structured exit questionnaire within 1 to 2 hours after each visit. For this study, a total of 24 SPs (7 females and 17 males) were recruited, trained, and hired as staff (see S1 Text for a further description).

In all scenarios in which the SP carried medical reports or images, the SPs conveyed to the provider that they did not know or understand what the reports showed—thereby varying the information available to the provider without altering the patient’s revealed beliefs or expectations. To assess the extent to which provider behavior is consistent across patients with identical presentations, 109 of the Case 1 interactions in Patna were completed at providers who had already completed a Case 1 interaction with a different SP actor.

Case 4 presents as an adult multidrug resistant (MDR) TB suspect with 4 weeks of cough and fever. The SP recalls receiving treatment from the government hospital in the past year for a similar condition and, if questioned, admits to not completing the TB treatment during the previous episode. All flags point towards TB recurrence, which should raise concern about drug resistance. Among 50 of the sampled Mumbai MBBS or higher providers, we randomly assigned SPs to present an experimental variant of Case 4, who carried the same TB-positive sputum AFB report as in Case 3. With this variant, we aimed to analyze the effect of a TB-positive diagnostic test signal by comparing quality of care provided to SPs portraying Case 4 with versus without the AFB report.

To facilitate outcome assessment for each interaction, labeled medicines and prescriptions given to the SPs were coded into 4 categories: anti-TB drugs, fluoroquinolone (FQ) antibiotics, other broad-spectrum antibiotics, or steroids. Coding was done independently by 2 doctors with expertise in TB (SS) and infectious diseases (RS). We chose to retain FQs as a distinct category of antibiotics because they can mask the presence of TB, rendering diagnosis more difficult, and can lead to delays in diagnosis [28]. Additionally, in Mumbai, providers often dispensed loose, unlabeled pills to the SPs. To properly identify these pills, we employed 2 pharmacists who worked independently to code them. On the basis of their assessments, we determined whether the variety of medicines given for each interaction included at least 1 broad-spectrum antibiotic and/or at least 1 steroid.

In addition to reporting the details of case management, each interaction was broadly classified as “correctly managed” or not, according to the STCI [29]. Taking a lenient approach, providers were not penalized for the use of unnecessary or even potentially harmful medicines, and thus the results presented are upper-bound estimates of quality, as measured by adherence to TB standards of care. The definitions of correct management are detailed in Table 2, and the levels of prescribing and dispensing medications are analyzed in the Appendix (S3 Text). S1 Fig elaborates on the “lenient” approach, illustrating the proportion of our “correct management” providers who would have been considered not correct if we had penalized other medications.

#### Analysis

We report raw proportions for outcomes of interest, with population mean estimates and CIs computed using inverse probability weighting. Based on the universe of private-sector providers listed from the mapping exercise in both cities (S1 and S2 Text), these weights are calculated such that each of the 8 city–case combinations contribute equally to overall estimates. Within each city–case combination, individual interactions are weighted based on the actual proportion of providers enrolled and not enrolled in the PPIA in that city’s provider sampling list within both the MBBS and non-MBBS strata versus the realized sample.

Therefore, the percentages reported for case management behaviors represent the estimated likelihood of the outcome occurring if a provider were chosen at random from the citywide population of providers rather than the percentage of interactions in our sample in which the behavior was observed. In addition to using these weights to estimate population likelihoods, we use them to calculate weighted odds ratios (ORs) in logistic regressions comparing variation in quality of care across provider types, city settings, and SP case scenarios.

In Supporting information (S3 Text), we present an ANOVA analysis, which was not prespecified, to determine how well our primary stratification characteristics explain variations in SP management. Among the sampled providers who received repeat Case 1 visits in Patna, we assess the level of consistency that those providers displayed across identical SP Case 1 visits using a different SP actor. We then use a quality proxy—checklist of history questions—to illustrate the amount of variation in provider behavior within each city–case combination.

In addition to the ANOVA analysis, we include the outcome of whether an SP was asked to return, which was not prespecified and requested during peer review. Considering that the data for this analysis are a subset of the data generated from a larger research project, we did not encounter any other deviations from the stated prospective analysis plan within the research protocol submitted to the ethics committees (S4 Text). All analyses and programs were written in Stata 14 (Stata, College Station, TX).

### Ethical approvals

Ethical approvals for this study were granted by the McGill University Health Centre in Montreal, Canada (REB No. 14-137-BMB) and the Subcommittee for the Ethical Approval of Projects at the Institute for Socioeconomic Research on Development and Democracy in Delhi, India. All SPs were hired as staff and received training and refresher trainings to protect themselves from potentially harmful events, such as injections during their interactions. For this study, a waiver of provider informed consent was sought with particular attention to the research ethics provisions under the Government of Canada Panel on Research Ethics, as well as a recent study by Rhodes and colleagues (2012) on ethical aspects of simulated patient studies commissioned by the US Department of Health and Human Services [30]. Supported by findings from the validation of the SP method for TB in urban India as reported in Das and colleagues (2015) [12], both ethics committees approved a waiver of provider informed consent in Mumbai and Patna because (1) the combination of informed consent and congregation of providers during association meetings and in the implementation of TB interventions that occurred during the study period posed threats to the scientific validity of the study objectives as well as to the risk of SP detection and (2) there is no more than minimal risk of participation to the SPs or providers [12]. Additional information on—including the rationale behind and approval of—the waiver of provider informed consent is detailed in S1 Text.

## Results

The results are presented in 3 sections. In the first section, we describe overall standards of care, focusing on correct case management, medicine use, and laboratory tests. In the second section, we document variation in the data by provider qualification and city. In the third section, we document variation across SP cases, focusing on the role of diagnostic certainty.

### Practice quality overview

**Fig 1:**
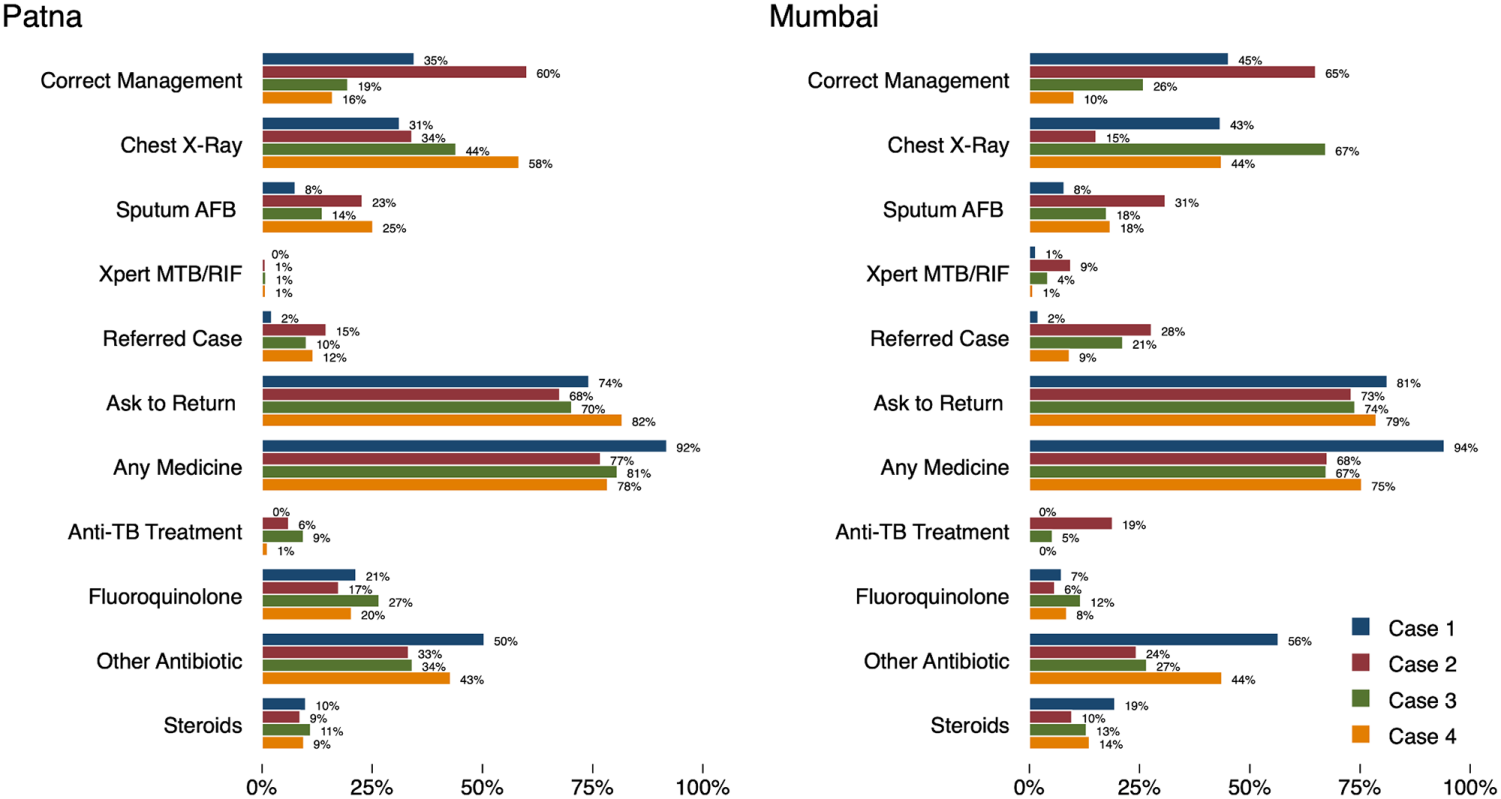
City-representative quality of care estimates. City-level estimates of quality of care for each of our case scenarios. These proportions represent the estimated frequency with which the action would be observed if the standardized case scenario was presented to a provider randomly selected from the sampling frame. These estimates are calculated using inverse probability weights corresponding to the sample frame as detailed in S2 Text for every city–qualification–PPIA–case combination in the data. *N* = 2,602. AFB, acid-fast bacilli; PPIA, Private Provider Interface Agency; TB, tuberculosis; Xpert MTB/RIF, Xpert Mycobacterium tuberculosis/Rifampicin, also known as GeneXpert.

In our study, 1,288 distinct provider practices were successfully visited by SPs at 1,203 health facilities across both cities. Among these providers, the majority were male (88%), had a clinic assistant (65%), and fell into the age category of 30–50 years (71%), which were all characteristics observed by SPs during the interactions. Fig 1 illustrates our main city-level estimates of average case management outcomes among the city-representative sample, with proportions estimated with weights to represent the estimated likelihood of the outcome occurring if a provider was visited by a patient at random from the citywide population of providers.

Among 2,602 SP interactions, 959 were correctly managed (35%; 95% CI 32%–37%), and 536 of 2,602 interactions (29%; 95% CI 26%–31%) had any diagnosis given to the patient. Among the 959 correctly managed interactions, 260 (37%; 95% CI 32%–42%) received any diagnosis. Among the correctly managed interactions, a greater proportion of providers ordered a CXR (677 of 959 [53%; 95% CI 48%–57%]) or referred the SP for further care (194 of 959 [36%; 95% CI 31%–40%]) than ordered a microbiological test for diagnosis (318 of 959 [31%; 95% CI 27%–36%]). Among the 194 referrals, 52% were to the private and 48% to the public sector.

Microbiological testing, including drug susceptibility testing (DST), was relatively infrequent across all case scenarios. Sputum smear testing was ordered in 389 of 2,602 (18%; 95% CI 16%–20%), sputum culture in 28 of 2,602 (2%; 95% CI 1%–3%), and Xpert MTB/RIF in 108 of 2,602 interactions (2%; 95% CI 1%–3%). For Case 2, when the SP carried an abnormal CXR suggestive of TB, 45 of 385 SP interactions (7%; 95% CI 4%–10%) resulted in any DST, while 99 of 385 were ordered a new CXR (25%; 95% CI 20%–30%). Similarly, of 486 interactions in which the SP presented with recurrent TB and suspected MDR-TB (Case 4), 26 were recommended any DST (3%; 95% CI 1%–5%).

Medicines were very frequently prescribed or dispensed, and we did not count the use of additional or unnecessary medications against the provision of correct management. At least 1 medication was prescribed or dispensed in 2,239 of 2,602 interactions (79%; 95% CI 76%–82%), with an average of 3.11 medications per interaction (95% CI 2.99%–3.23%). Across all 2,602 interactions, broad-spectrum antibiotics other than FQs were given in 1,227 (39%; 95% CI 36%–42%), FQ antibiotics in 328 (15%; 95% CI 13%–17%), and steroids in 164 interactions (6%; 95% CI 5%–7%).

The use of anti-TB medications was minimal and was mostly limited to MBBS-qualified providers. Anti-TB medications were prescribed in 118 of 2,602 interactions (5%; 95% CI 4%–6%), with MBBS or higher providers accounting for 112 of those 118 instances. Among the 118 TB prescriptions, 113 included correct isoniazid, rifampicin, pyrazinamide and ethambutol (HRZE) prescriptions—1 of these additionally included the second-line drug clofazimine, and 2 also included streptomycin. Among the 5 instances that did not include HRZE, 4 were HRE, and 1 was the second-line medication cycloserine prescribed alone.

In contrast with correctly managed interactions, interactions in which correct case management was not observed did not demonstrate any kind of consistent “alternate” protocol that could theoretically be justified in a polluted urban environment that can result in non-specific respiratory symptoms like cough. Given a patient with a 2- or 3-week cough in a polluted city like Patna, providers could, for example, be observed to adopt a “wait-and-see” approach by offering palliative symptomatic care and asking the patient to return after a few days, even though this practice technically falls short of the international and national standards of TB care we used to benchmark management in this study.

**Fig 2:**
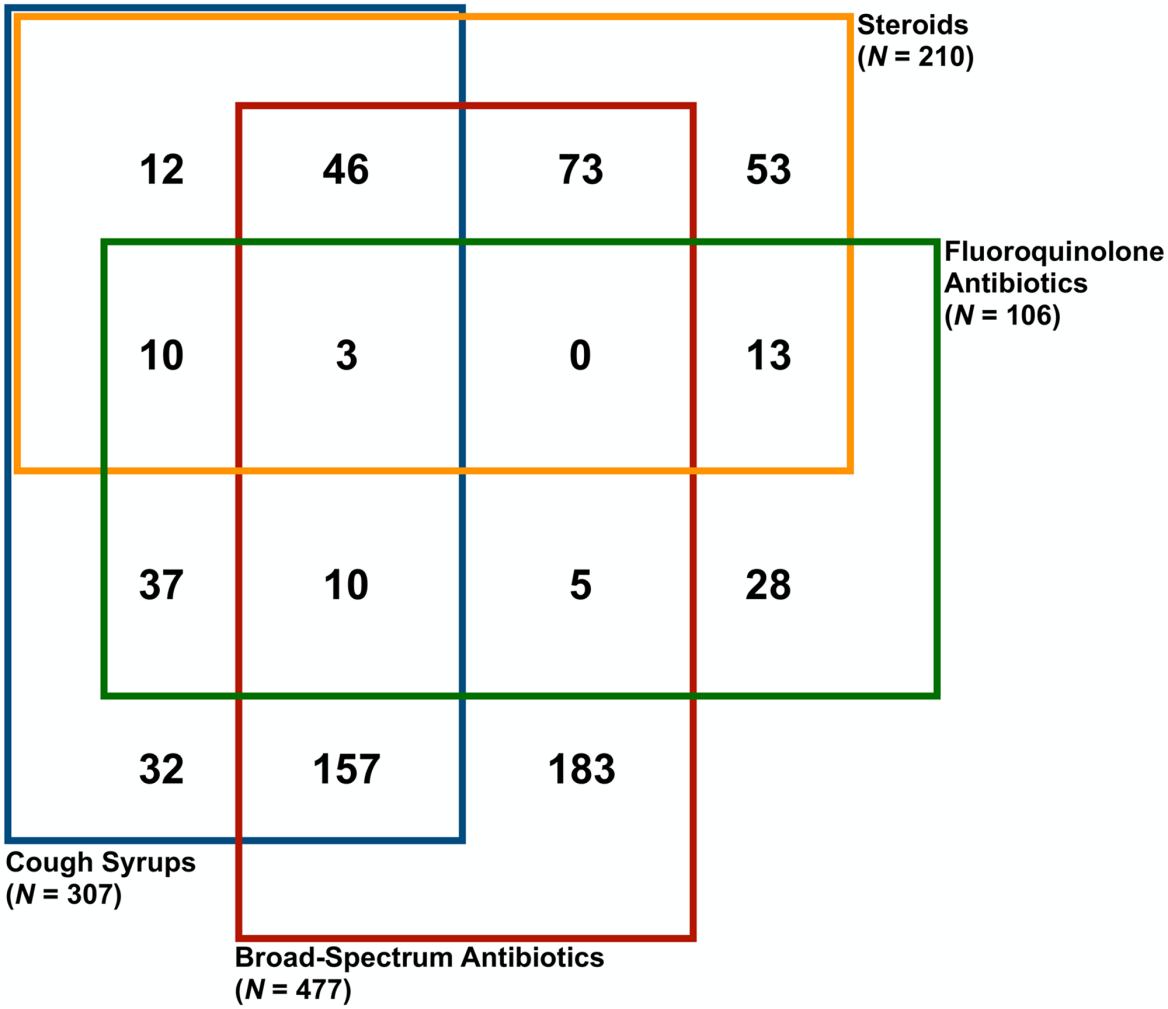
Management of Case 1 when no correct treatment was given. Frequency in which Case 1 was managed with possible combinations of steroids, cough syrups, broad-spectrum antibiotics, and FQs, when no correct management was given. There were *N* = 834 Case 1 interactions that did not meet the criteria for correct management, and 172 interactions resulted in none of these case management behaviors. FQ, fluoroquinolone.

We do not observe this type of concentration on a single alternative protocol in our data. For example, of the 834 Case 1 interactions that were not correctly managed (Fig 2), 183 (22%) received only a non-FQ broad-spectrum antibiotic; 294 (35%) received a non-FQ broad-spectrum antibiotic as well as an FQ, steroid, and/or cough syrup; 185 (22%) received 1 or more of those medications without a non-FQ broad-spectrum antibiotic; and 172 (21%) received something else entirely (or nothing at all).

### Variation by qualification and setting

**Fig 3:**
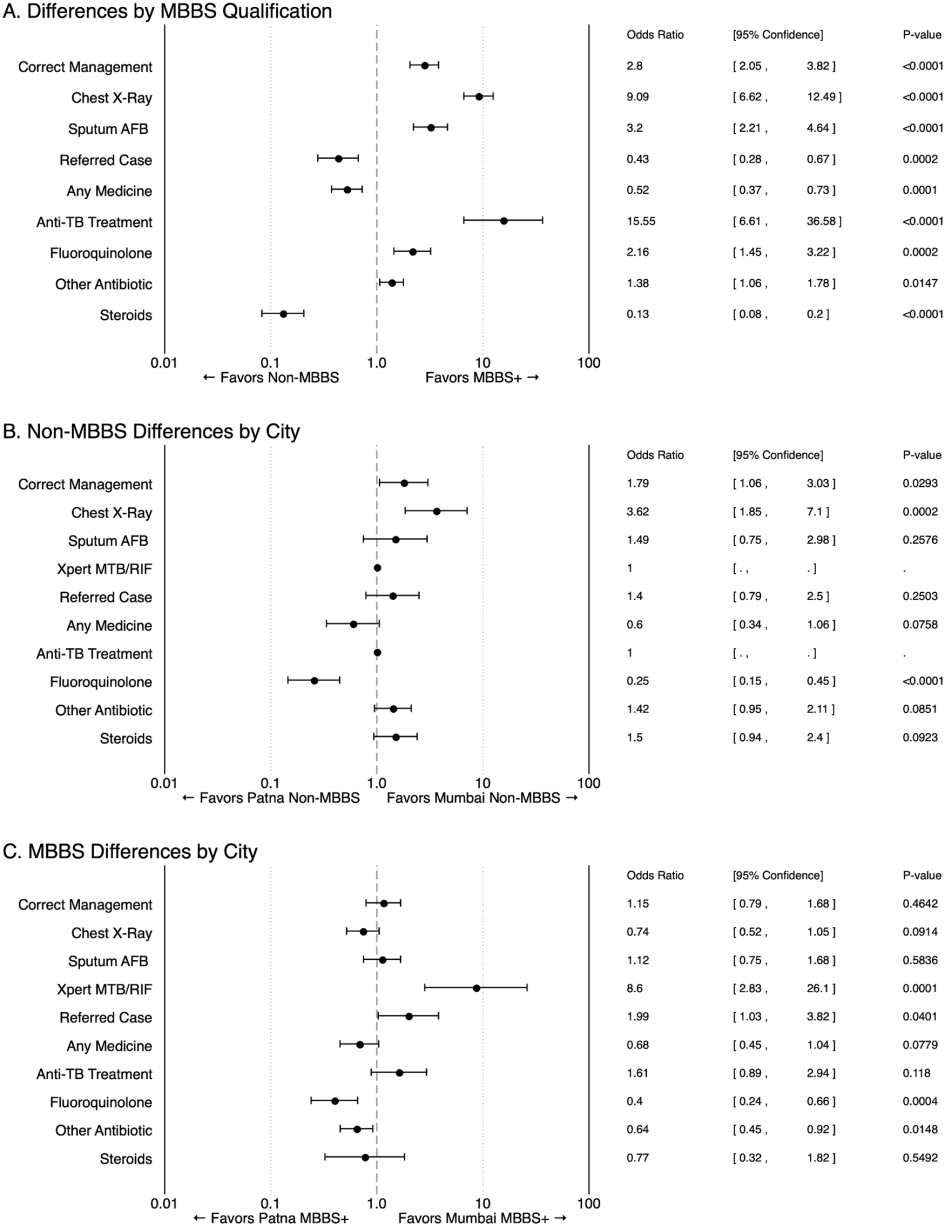
Quality of care differences by provider qualification and location. Estimated ORs between various groups of providers, for the frequency in which the indicated management action is observed across all case scenarios. Panel A reports differences by MBBS qualification level, pooled across all observations. This regression includes controls for city setting and case scenario (*N* = 2,602). Panels B and C report similar ORs estimated across cities, stratified by MBBS qualification (*N* = 1,448 and 1,154, respectively). These regressions include controls for case scenario. AFB, acid-fast bacilli; MBBS, Bachelor of Medicine, Bachelor of Surgery; OR, odds ratio; TB, tuberculosis; Xpert MTB/RIF, Xpert Mycobacterium tuberculosis/Rifampicin, also known as GeneXpert.

One potential explanation is that the wide variation in management reflects systematic practice variation by qualification and/or setting, and we turn to this hypothesis next. Fig 3 reports ORs for differences in quality of care outcomes by qualification (top panel) and by city stratified by qualification (middle and bottom panels). MBBS providers, who make up 58% of all providers in the Patna sampling list and 50% of all providers in the Mumbai list, were more likely than non-MBBS providers to correctly manage cases (weighted OR 2.80; 95% CI 2.05–3.82; *p* < 0.0001), ask for CXR and/or sputum tests, and initiate anti-TB treatment. Despite providing relatively higher-quality care, MBBS providers only correctly managed 709 of 1,304 interactions (54% of interactions; 95% CI 52%–57%). MBBS providers were also more likely than others to prescribe unnecessary or harmful antibiotics, including FQs, although their use of steroids was notably lower.

Additional analysis in S6 Fig shows that the differences between AYUSH and other non-MBBS providers in Patna, where both practice, were surprisingly small. In fact, AYUSH providers were less likely than others to manage or refer cases according to STCI, although the others gave more unnecessary antibiotics. Use of nonallopathic medications was also diverse. For example, although most non-MBBS providers in our Mumbai sample are AYUSH, only 63 of 996 non-MBBS SP interactions in Mumbai (6%; 95% CI 4%–8%) involved explicitly labeled homeopathic or Ayurvedic medicines. By contrast, non-MBBS providers in Patna used labeled Ayurvedic or homeopathic medicines in 113 of 302 interactions (41%; 95% CI 33%–49%).

Differences in case management were smaller across the 2 cities. Across all cases, Mumbai non-MBBS providers were more likely than Patna non-MBBS providers to correctly manage cases (weighted OR 1.79; 95% CI 1.06–3.03; *p* = 0.0293), primarily by ordering CXR (weighted OR 3.62; 95% CI 1.85–7.10; *p* = 0.0002). Most other behaviors were not significantly different across the cities. MBBS-qualified providers were even more similar across cities in terms of correct management (weighted OR 1.15; 95% CI 0.79–1.68; *p* = 0.4642), with the notable exception that Mumbai MBBS providers utilized Xpert MTB/RIF testing much more frequently than MBBS providers in Patna (weighted OR 8.60; 95% CI 2.83–26.10; *p* = 0.0001). Both MBBS and non-MBBS providers in Mumbai were less likely to prescribe FQs than Patna providers (weighted OR 0.4; 95% CI 0.24–0.66; *p* = 0.0004, and 0.25; 95% CI 0.15–0.45; *p* < 0.0001, respectively), and the dispensing or prescribing of steroids and broad-spectrum antibiotics did not differ systematically across the 2 cities.

ANOVA decomposition (S3 Fig) shows that our primary stratification characteristics of qualification and setting typically predicted less than 25% of the observed variation in each of our primary case management outcomes with the exception of correct case management and ordering a CXR (for which 25%–50% of observed variation was predicted). By contrast, in a subsample in which 109 Patna providers received a repeat Case 1 interaction (S4 Fig), consistency levels between the 2 visits were near 75% for all behaviors. Therefore, we observe that practice is highly variable across providers, but setting and qualification strata can explain only a fraction of the wide variety in management practices.

Using essential-history checklist completion as a proxy for the distribution of individualized quality levels (S5 Fig), our results suggest that there is a substantial and unexplained idiosyncratic component to quality that varies widely even within each city and qualification level. Rather than tight clustering around the group means, in all cases, we observe a “fat tail” of both low- and high-quality MBBS and non-MBBS providers in each city. Thus, moving beyond averages to full distributions of quality yields important and nuanced additional results for the estimation of “average” outcome quality, both by city and by qualification.

### When do providers perform better? Variation by SP case

The final source of variation we assess is the SP case presentation itself. In our previous work [14], we found that pharmacists were more likely to correctly manage a case if SPs carried a TB-positive diagnostic test, even as the SP made it clear that she did not know what the results of the test meant. The SP cases presented in this study reflect a similar design, with the varied presentation intended to exogenously adjust the providers’ initial degree of certainty about the patient’s true diagnosis. When the SP carried either an abnormal CXR (Case 2) or an AFB-positive sputum report (Case 3), providers were observed to be more likely to order tests consistent with a suspicion of TB compared to the Case 1 presentation.

In Case 2, in which the SP carried an abnormal CXR, a second CXR (which would ostensibly provide no new information) was ordered in 99 of 385 instances (25%; 95% CI 20%–30%). In Case 3, in which the SP carried a TB-positive sputum AFB report, 208 of 354 were recommended a CXR (56%; 95% CI 49%–63%), and 54 of 354 were ordered a new AFB smear (16%; 95% CI 11%–21%). In terms of inappropriate behaviors, variation across SP case had some impact on the use of inappropriate medications, but it did not reduce it anywhere close to 0 in any case (S1 Fig). Prescribing unnecessary medicines ranged from 284 of 385 instances in Case 2 (72%; 95% CI 67%–77%) to 1,290 of 1,377 instances in Case 1 (93%; 95% CI 91%–95%). Use of broad-spectrum antibiotics ranged from 105 of 385 Case 2 interactions (29%; 95% CI 23%–34%) to 799 of 1,377 Case 1 interactions (53%; 95% CI 50%–57%), and use of FQs ranged from 43 of 385 Case 2 interactions (12%; 95% CI 8%–16%) to 52 of 354 Case 3 interactions (19%; 95% CI 13%–25%).

**Fig 4:**
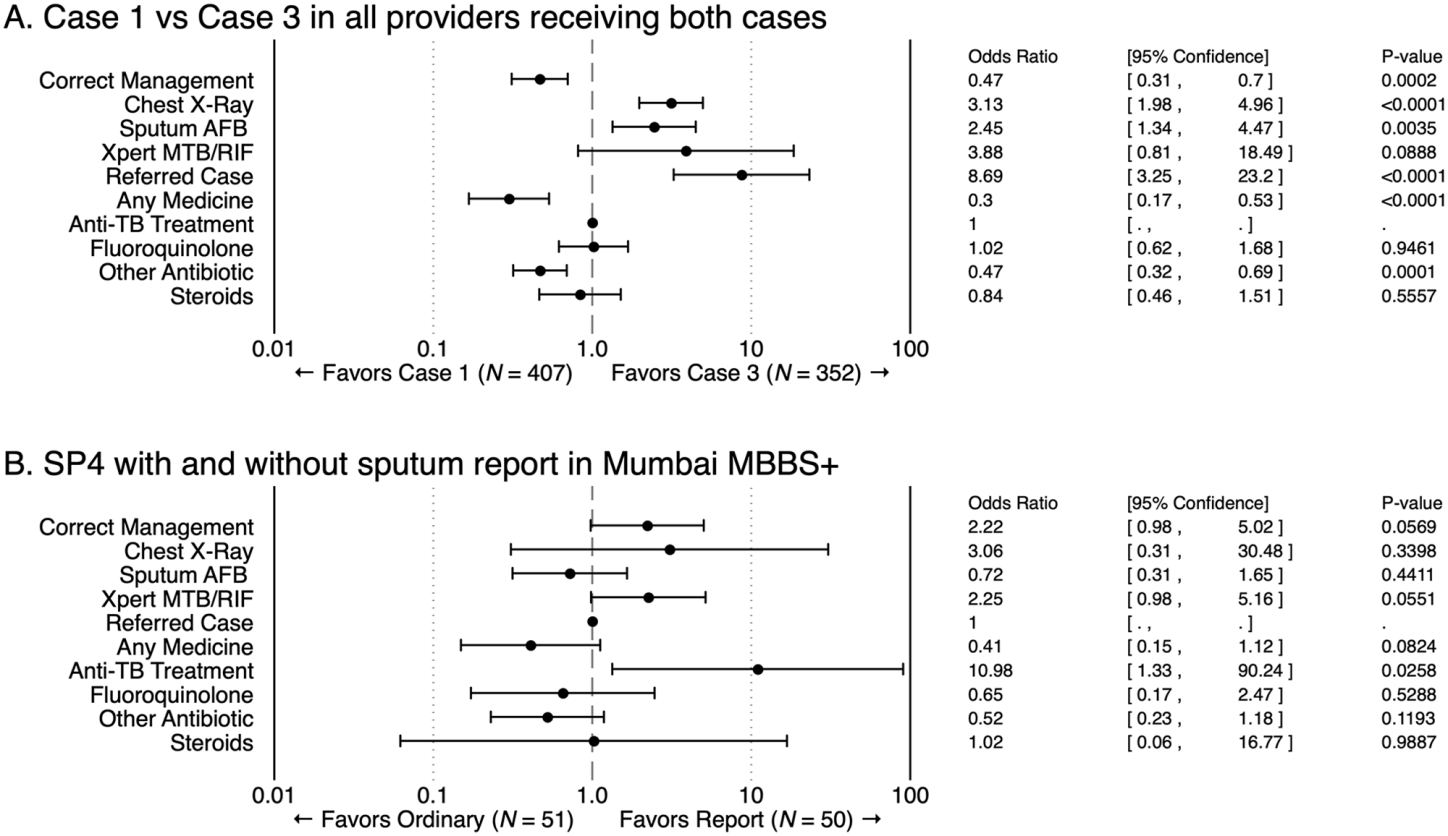
Quality of care differences between SP case scenarios. Estimated ORs between specific case scenarios for the frequency with which the indicated management action is observed. Panel A reports estimated ORs between Case 1 and Case 3, including only those providers who received both cases (*N* = 759 interactions). Panel B reports estimated ORs between Mumbai MBBS providers who received the experimental version of Case 4 that carried the same sputum report against a comparable sample who received the ordinary Case 4 presentation (as described in S1 Text; *N* = 101 interactions). AFB, acid-fast bacillus; MBBS, Bachelor of Medicine, Bachelor of Surgery; OR, odds ratio; SP, standardized patient; TB, tuberculosis; Xpert MTB/RIF, Xpert Mycobacterium tuberculosis/Rifampicin, also known as GeneXpert.

Fig 4 highlights selected cross-case variation using logistic regressions. The figure reports estimated differences between Case 1 and Case 3, using only providers who received both cases. It also reports differences observed in Case 4 against our alternate version of Case 4 carrying the same TB-positive sputum AFB report at identically randomly sampled MBBS-qualified providers in Mumbai, in which the variation in diagnostic certainty is causally identified through the use of random assignment of the AFB report in an otherwise-identical case presentation.

Carrying a positive sputum report in Case 3 was associated with an increase in the frequency of CXRs (OR 3.13; 95% CI 1.98–4.96; *p* < 0.0001) and sputum AFB tests (OR 2.45; 95% CI 1.34–4.47; *p* = 0.0035), as well as a nonsignificant increase in Xpert MTB/RIF tests (OR 3.88; 95% CI 0.81–18.49; *p* = 0.0888) and referrals (OR 8.69; 95% CI 3.25–23.2; *p* < 0.0001) compared to the same providers’ behavior in the Case 1 interactions. In the experimental comparison in which only the diagnostic information was varied for SPs portraying Case 4 while carrying versus not carrying a TB-positive sputum report (S1 Text), we use the fact that the report was randomly assigned as indicative of causal impact. Among the 50 instances in which Case 4 SPs carried a sputum report, the provider saw the report in 45 instances (90%) and asked detailed questions about the past treatment in 38% of interactions. The results are broadly similar although nonsignificant: when the Case 4 SP carried the sputum report, correct management increased from 29% to 48% (OR 2.22; 95% CI 0.98–5.02; *p* = 0.0569), largely due to greater provider use of appropriate DST. Providers’ use of medication also decreased from 86% to 72%, though this was not significant (OR 0.41; 95% CI 0.15–1.12; *p* = 0.0824). However, quality improvements were not consistent across all dimensions: the use of first-line anti-TB medicine, which is not considered correct treatment for a suspected drug-resistant case and could contribute to greater drug resistance, increased from 2% to 18% when the report was presented (OR 10.98; 95% CI 1.33–90.24; *p* = 0.0258).

## Discussion

TB is a persistent health challenge for India and is one of the top 5 causes of death between the age of 30 to 69 [31]. With India’s goal of eliminating TB by 2025 as stated in the NSP, the success of this plan heavily depends on whether India’s large, unregulated, and diverse private sector can be effectively engaged to identify missing patients with TB and ensure that all patients with TB receive quality TB care [32].

This study extends the evidence from our pilot study that validated the use of the SP method for assessing TB quality of care in an urban India setting [12] and from our research on TB-management practices of pharmacists assessed with SPs across 4 Indian cities [13,14]. Our validation study assessed quality of TB care from a purposive provider sample, and with this study, we were able to further apply the SP method for TB to analyze representative levels and variation of quality among MBBS and non-MBBS providers in the private health sector of 2 cities. In addition, 2 micro-experiments allow us to better understand the drivers of quality of care in this setting.

Our city-representative study shows significant deficits in the average provider’s management of TB cases in both study cities. This low quality is characterized by underuse of appropriate diagnostics and widespread use of unnecessary medications, including antibiotics and contraindicated FQs. Even though MBBS-qualified providers managed the SP cases better on average, there was still considerable variation within qualification in each setting and relatively little difference between the 2 cities on average. We further observed that TB-specific management increased with diagnostic certainty. We also present suggestive evidence that specific providers adhere consistently to an idiosyncratic protocol when faced with repeated identical cases. Because providers are making the same mistakes consistently, the SP method provides novel information to TB-control programs on the specific actions that need to be improved.

Representing the quality variation during the first year of city-wide private-sector engagement efforts, our data further underscore the need to work with the private sector to improve quality of TB care. These study results, complemented with the 4-city analysis on pharmacist behaviors, provides new multicity, qualification-specific information for TB control in India. It remains to be seen the extent to which private-sector engagement efforts—such as the PPIA pilots—will make an impact on quality and be able to sustain improvements in quality of care. Incorporating quality measures alongside program implementation is the first step, particularly as aspects of the PPIA model are being scaled up in more than 40 Indian cities, supported by the Global Fund.

We emphasize that the low observed proportions of correctly managed interactions do not fit a hypothesis in which providers followed an alternate protocol that reflects “what is good for the patient but not for society” (such as a wait-and-see approach for a patient with persistent cough). Providers treated the SPs in idiosyncratically different ways without a consistent protocol. Neither are the data consistent with the view that very high patient loads are responsible for low quality. Of the weighted 2,602 interactions, 45% had no other patients waiting, 65% had a queue of 1 or fewer, 75% had 2 or fewer, and 95% had 10 or fewer. This is similar to what has been observed in previous SP studies as well as time-and-motion studies in health clinics [33].

We consider, instead, the following 2 broad classes of explanations for this behavior: (a) providers have a difficult time diagnosing TB appropriately, and (b) private providers deviate from established standards for financial gain. There is evidence for both types of behavior, implying that quality deficits are not driven by either knowledge gaps or financial incentives alone. In favor of an explanation driven by poor diagnostic skills, we find that improving certainty about the diagnosis had a positive effect on quality of care (although the results are not statistically significant in the Case 4 comparison because of the small sample size, they are quantitatively as large as those in the Case 1 to Case 3 comparison). However, lack of diagnostic certainty was not the only indicator of poor quality we observed in our study, nor did quality improve across every dimension when the test results were provided to the provider. Increasing diagnostic certitude improved correct case management but had smaller effects on reducing inappropriate medicine use.

This suggests that financial considerations and poor diagnostic ability may both play a role in explaining the patterns we observe and mirror previous findings with pharmacists in 4 urban Indian locations [14,34]. Across settings, we find that these diagnostic practices are highly predictive of whether the provider offers correct management in a given interaction, but we cannot “force” providers to take detailed histories from their patients because a good history and physical is an essential indicator of quality in and of itself.

Our study has several strengths and contributes to the literature in several ways. First, we representatively sampled large numbers of private health providers in 2 Indian cities, and after weighting to the city universe of providers, we provide precise estimates of provider behavior at the city level. Because the analysis is representative of these 2 Indian cities, caution is warranted when generalizing to a context outside of urban India. Second, by using unannounced SPs, we captured actual provider behavior, as opposed to self-reported knowledge or practices. Given our prior work showing a big “know-do gap” (the gap between what providers know and what they do in actual practice) [12], the SP methodology better reflects reality for patients than any other existing method used to measure quality of care. Third, our study included MBBS-qualified providers, as well as AYUSH providers and those with other or no qualifications, capturing the complexity of training within the Indian private healthcare sector. Fourth, by developing 4 different SP case presentations, we studied how providers dealt with various stages of TB disease and varying levels of diagnostic certainty. Lastly, by assessing outcomes by city, provider qualification, and type of case, we assessed the most important sources of variation in quality of care. While previous studies showed suboptimal quality of care, our study was able to explore the role of provider qualifications on quality using 2 city-wide, representative samples.

Our study has limitations. First, because we do not observe how patients actually choose providers, patient sorting by qualification, geography, personal relationships, price, reputation, or other unobserved signals of quality prevents extrapolation to the likely outcomes for actual patients with TB. This remains an important area for future work. Second, our cases are designed as one-time interactions, and the SP data do not reflect follow-up visit pathways, which have been shown by other studies to be long and convoluted and had various forms in our data (S2 Fig). For instance, we cannot say, from this study, what the doctor would do after the patient has returned after completing a CXR as ordered. To the extent that doctor behavior is different when the patient comes with a CXR that the doctor herself recommended (rather than with a CXR ordered by another doctor), our approximations of provider behavior under different scenarios may be erroneous. The ability for the SP method to measure quality of care measures in follow-up visits with the same SP individual in similar settings has not been published to our knowledge, and—given the frequency of providers asking for patients to return exhibited in this study—it could be worthwhile to explore the potential for the method to assess whether the likelihood of receiving better care increases when a patient returns. With this, it would be important to understand the extent to which real patients return upon a provider’s request, as well as the necessary work needed so that SPs are not detected.

While not a limitation per se, we also highlight that our definition of correct case management follows national and international TB standards of care. We have chosen to use these definitions because they allow for comparability across studies and disciplines our analyses of the data using a clearly prespecified protocol. But our data could also raise questions about the viability and validity of these standards. For instance, it is reasonable to ask whether—in cities with air pollution, which can cause respiratory symptoms—healthcare providers should be asked to send a patient with a 3-week cough for a sputum test rather than giving them symptomatic therapy and asking them to follow up as required.

Our data are not consistent with the idea that providers were following a single alternate protocol because virtually every combination of drug classes was used for the SP cases. Nevertheless, there could be justifiable reasons why providers still choose to deviate from established standards of care. One way, then, to interpret our findings and our use of the term “correct case management” is as an ordinal ranking rather than adherence to a standard: providers who follow the standards are of higher quality, but this does not imply that those who do not follow the standards necessarily provide “incorrect” care. Instead of imposing alternate standards, we have chosen to present the full set of management practices for each SP case to allow readers to make their own more nuanced judgments. We have also made the data publicly available so that researchers can simulate correct management under alternate standards, in turn stimulating debate around whether these standards themselves require further revision.

Despite the limitations, our large-scale, 2-city quality of care study provides accurate and representative estimates of provider behavior that may inform not only quality-improvement efforts in health but also interventions to improve TB care and reduce transmission in the community.

## Supporting information

S1 Text Fieldwork details. Description of SP case scenarios; SP recruitment, script development, and training; rationale for approved waiver of provider informed consent; provider sampling; assignment of SP cases to providers. SP, standardized patient.
(PDF)

S2 Text Statistical methods. (PDF)

S3 Text Supplementary results. Assessing variation in provider management of cases; ANOVA decomposition of explained variance; consistency of individual providers across visits; distributions of providers within city-qualification groups.
(PDF)

S4 Text Pre-analysis plan from study protocol. Study design and methods, and data analysis plan.
(PDF)

S1 Fig Correct management of SP case scenarios, with alternate definitions. SP, standardized patient.
(PDF)

S2 Fig Follow-up requests from providers by case management outcome, with alternate definitions. (PDF)

S3 Fig ANOVA decomposition of quality correlates. (PDF)

S4 Fig Consistency of providers in repeated Case 1 visits. (PDF)

S5 Fig Distributions of checklist completion in Case 1 visits. (PDF)

S6 Fig Comparison between AYUSH and non-AYUSH treatment outcomes. AYUSH, Ayurveda, Yoga, Unani, Siddha, or Homeopathy.
(PDF)

S7 Fig Sampling flowcharts by primary strata. (PDF)

S1 Checklist STROBE statement. (PDF)

## Abbreviations

AFB: acid-fast bacillus
AYUSH: Ayurveda, Yoga, Unani, Siddha, or Homeopathy
CXR: chest X-ray
DOTS: directly observed treatment, short course
DST: drug susceptibility test
FQ: fluoroquinolone
HRZE: isoniazid, rifampicin, pyrazinamide and ethambutol
INR: Indian rupee
ISTC: International Standards for TB Care
MBBS: Bachelor of Medicine, Bachelor of Surgery
MDR: multidrug resistant
NSP: National Strategic Plan
OR: odds ratio
PPIA: Private Provider Interface Agency
RNTCP: Revised National TB Control Programme
SP: standardized patient
STCI: Standards for TB Care in India
TAG: Technical Advisory Group
TB: tuberculosis
Xpert MTB/RIF: Xpert Mycobacterium tuberculosis/Rifampicin
