# Supplementary material for: Variations in the quality of tuberculosis care in urban India: A cross-sectional, standardized patient study in two cities
Source: PLoS Med. 2018 Sep 25;15(9):e1002653. doi: 10.1371/journal.pmed.1002653 (PMC6155454; doi:10.1371/journal.pmed.1002653)
Supplement: S1 Text — Description of SP case scenarios; SP recruitment, script development, and training; rationale for approved waiver of provider informed consent; provider sampling; assignment of SP cases to providers. SP, standardized patient. (PDF) [file pmed.1002653.s001.pdf]

S1 Text: Fieldwork Details

S1a Description of SP Case Scenarios

Four tuberculosis (TB) case scenarios were developed to document the level and variation in quality of care for TB among sampled providers. For each case, both the clinical case presentation and social contexts were developed and agreed upon by a technical advisory group, which included clinicians, economists, anthropologists, experts in international and national TB guidelines, and other stakeholders. The four cases were:

1. **Case 1 (Naïve TB Suspect)** – A classic case of presumptive TB with 2-3 weeks of cough and fever. The SP presents to the providers and begins the interaction with the opening statement: "Doctor, I have a cough that is not getting better and some fever too."

2. **Case 2 (TB Suspect with Abnormal Chest X-Ray)** – A classic case of presumptive TB who has had 2-3 weeks of cough and fever, a history of completed chest X-ray and 1 week of broad-spectrum antibiotic treatment ordered by another provider, with no improvement. The SP carries a digital chest X-ray dated within the last 10 days with evidence of abnormalities, and the blister pack of amoxicillin with him/her. The SP begins the interaction by saying: "Doctor, I have had cough and fever. It is not getting better, even though I went to a doctor and took medicines also."

3. **Case 3 (TB Case)** – A chronic cough with a positive sputum smear report for TB from a public health facility. The SP carries the sputum microscopy test report and displays it prominently on his/her lap, mentioning that he/she has had his/her sputum tested. The SP begins the interaction by saying: "I am having a cough for almost a month now and also have a fever. I visited the Government hospital, and they gave me some medicines and did sputum tests."

4. **Case 4 (MDR Suspect)** – A classic case of presumptive TB with 2-3 weeks cough and fever, and, if asked, a history of previous incomplete TB treatment, which would raise the suspicion of multi-drug-resistant TB. The SP begins the interaction by saying: "Doctor, I am suffering from a bad cough. One year ago, I got treatment in the Government hospital, and it had got better, but now have a cough again. I went back to the same hospital, and they did a sputum test." In 50 additional interactions in Mumbai, the SP is randomly assigned to carry a TB-positive sputum test report as in Case 3 and displays it prominently on his or her lap.

S1b SP Recruitment, Script Development, and Training

SP Recruitment

For the two-city study presented here, 24 individuals (8 females and 16 males) were recruited and hired as SPs, including both new recruits and individuals. The SP cohort in each city was comprised of a different set of individuals with 13 of the 24 individuals hired for the study in Patna and 17 of the 24 individuals hired as SPs for the study in Mumbai. Some SPs had prior experience as they had participated in our validation study in Delhi (Das et al. 2015) and/or other SP studies assessing quality for other health conditions aside from TB.

During the recruitment process, all potential SPs underwent a health screening questionnaire and checkup, and all SPs in the final cohorts were seemingly healthy, which meant they had no apparent health conditions that could confound the case presentation and interaction with providers. The SPs, although recruited specifically to fit each case scenario and corresponding narrative, differed in age, gender, height, and weight. The average age of all the SPs was 30. The youngest was 21; the oldest was 39. The 16 males weighed 50 to 74 kilograms and were 160 to 184 centimeters tall. The 8 females weighed 46 to 72 kilograms and were 147 to 160 centimeters tall.

For Patna data collection (21 November 2014 to 28 February 2015), the SP cohort consisted of 13 individuals (5 female), who conducted interactions with MBBS or

higher and non-MBBS providers. For these interactions 6 SPs (2 female) were Case 1, 2 SPs (1 female) were Case 2, 3 SPs (2 female) were Case 3, and 2 SPs (1 female) were Case 4. All SPs in Patna conducted some Case 1 interactions, regardless of their primary assignment. All SPs were originally from the State of Bihar, of which Patna is the capital.

In Mumbai, 17 individuals (5 female) conducted fieldwork between 2 April 2015 to 21 August 2015. For these interactions, 8 SPs (2 female) were Case 1, 2 SPs (1 female) were Case 2, 3 SPs (1 female) were Case 3, and 4 SPs (1 female) were Case 4. SPs were originally from the States of Bihar (5), Madhya Pradesh (1), and Maharashtra (11). Primary languages spoken by the SPs included: Angika (1), Bangali (1), Bhojpuri (1), Hindi (4), Magahi (2), and Marathi (8).

SP Script Development [1]

Each SP case scenario described in S1a was coupled with a script. Each script is a narrative that describes the social and family contexts of the patient. The scripts were developed under the guidance of an anthropologist (VD) with active supervisor and SP participation. Together, the case scenarios and scripts were piloted in our validation study in Delhi as presented in Das et al. (2015). They were again refined based on field and data management lessons from Delhi and again during and after training in Patna and Mumbai for the study presented in this paper.

The two most important considerations for script development that were also tightly linked to SP training were: First, the clinical symptoms and case history had to reflect the social and cultural milieu of which the SP was assumed to be a member, and second, the presentation of symptoms and answers to history questions had to be consistent with biomedical facts about the disease.

On the former, SPs brought a lot of socially appropriate understanding of the local vocabularies through which symptoms were to be presented and also about typical life histories that would correspond to the age, sex, caste, religion and class

[1] The following text is an edited version of the Supplemental Appendix from Das et al. (2015), tailored for this study.

of the character that the SP was portraying. As a simple but crucial example, people among the strata the SPs were drawn from do not often use thermometers to measure temperature but report fever on the basis of the sensation of heat and rapid pulse. The inputs by SPs in script development were crucial from this perspective.

The latter issue was to train SPs to present symptoms and answer questions pertaining to case history that were medically correct. For example, all opening statements and questions pertaining to the type of cough and its duration were standardized. A critical part of the training was to help SPs distinguish between questions to which answers could be improvised but had to be appropriate to the social role of the SP and answers that had to be given using local idioms but in a standardized format without any alterations.

The dual aim of presenting the disease in a manner that was not misleading and avoiding detection were largely successful because the reasoning behind both objectives was carefully and repeatedly explained to the SPs and because of their active involvement in the script development and hands-on training. SP case scripts are available from the authors upon request.

SP Training [2]

To portray the four SP cases, the 24 otherwise healthy individuals recruited as SPs were trained in each city to finalize the case presentation given their knowledge of context, internalize the scripts and cases, be able to debrief with a supervisor within 1-2 hours of the interaction, and present in clinical settings in a way that would avoid any potentially harmful risks and detection. Thus, SP training was designed with four specific aims:

1. SPs correctly present the cases in a standardized way;

2. SPs accurately recall the interaction that occurred with health care providers;

3. SPs avoid both detection or any suspicion that the interaction was not genuine; and

[2] The following text is an edited version of the Supplemental Appendix from Das et al. (2015), tailored for this study.

4. SPs are prepared to avoid potentially harmful risks that can occur to them.

The first two aims were achieved through extensive classroom training in case presentation and recall. Classroom training was complemented with mock interviews and followed by live supervised dry runs in the field at clinics not sampled for the study. Our pilot study in Delhi described in Das et al. (2015) also included the use of tape recorders in a selected subset of interactions, which we used to verify that the results reported on the structured questionnaires were accurate reflections of the clinical interactions.

For the third aim, SPs were carefully instructed to avoid detection by the following methods. First, our recruitment strategy ensured that SPs came from low-income areas or slums from the same cities in which the project was located, so they could easily pass for genuine and local patients, but the areas from which they came were located far from the field sites, so they would not be personally known in the areas they conducted interactions. Second, supervisors for SP fieldwork traveled into the field for 'scoping exercises' before any of the SP interactions were conducted. These scoping exercises helped supervisors to familiarize themselves with landmarks, clinic locations and addresses, general setting, operating hours, length of potential wait time or queues, need for clinic appointments, and other potential issues that could pose challenging to the SP interaction. Based on scoping, the team ensured that SPs were set up to conduct as-successful-as-can-be interactions. Third, during the training, time was organized such that SPs could internalize the characters for each case and the details of their mock stories through which the character was made alive to them. In mock interviews during training, supervisors added unscripted questions with regard to the patient's family or neighborhood details, which SPs were able to answer spontaneously because they were of the actual social background that was being approximated in the characters they were portraying. Finally, dry runs were conducted in which the supervisor was present in the shop on the pretense of buying something, such as toothpaste or an over-the-counter cough syrup, and thus could watch the interaction and use additional training time to improve the SPs'

presentations of the cases.

For the fourth aim, SPs participated in active discussions on risk mitigation strategies. Together with the supervisors, the SPs brainstormed what they could do to mitigate risks or avoid situations that could be abnormal. They were then extensively trained on these and additional risk mitigation strategies. (Communication on these matters were prioritized and extended into the data collection period. Throughout the data collection period, which was weathered with fog that reduced visibility to several meters, cold temperatures, scorching heat at times above 43°C/110°F, high humidity, annual monsoons, flooded roads, medicine bans, and elections, supervisor and SP meetings would occur near weekly. These meetings provided additional opportunities to discuss potential issues as a team and how to mitigate them as they were encountered in the field.)

In Patna, SPs were trained initially to present various cases to chemists, and after the completion of chemist interactions, an additional 5-day training was conducted for all the SPs to train them for Cases 1-4 and the associated risk mitigation techniques. Dry runs were conducted in both cities for interactions with providers. The dry runs also provided an opportunity to test the chest X-rays (CXRs) and corresponding reports carried by Case 2 and for the sputum reports carried by Case 3. Different sets of abnormal CXRs for both females and males were procured by ISERDD from a contact of a member of the QuTUB team (SS). The digital CXRs were shipped from Hyderabad throughout fieldwork approximately every ten days. Sputum reports were researched and produced by the field team.

In Mumbai, all of these individuals took part in a comprehensive and rigorous 10-day training, which included 3 days of dry runs. The entire standard training was done in Hindi, since even Marathi-speaking individuals were expected to encounter doctors speaking in Hindi, and after classroom training, Marathi-speaking SPs went over scripts and exit questionnaires in Marathi, followed by dry runs in Marathi. Dry runs were conducted outside of the wards selected for the sample. There were several full meetings for SPs and supervisors, and refresher trainings were conducted

every time a new schedule was provided to the field team, approximately every three weeks.

S1c Rationale for Approved Waiver of Provider Informed Consent

Ethics guidelines on health service audit studies state that SPs should be used in cases where the person being sent the SP is providing a service to other people and where other options have been carefully studied but cannot answer the research questions required. In addition, there should be minimal risk to the participants. Based on 10 years of research, we have demonstrated the difficulties of obtaining quality of care data without using SPs (Alderman et al., 2014). In previous SP studies, we have requested and received waivers of informed consent from ethics committees at Johns Hopkins University, Harvard University, and Duke University. Another SP study conducted at the Universidad Peruana Cayetano Herediahas has also received waiver of informed consent. These waivers have been granted under the provisions for waiver or alteration of the informed consent requirements under the United States Department of Health and Human Services regulations 45 CFR 46.116(d) [3].

Although the ideal study design would include informed consent, we sought a waiver because (a) we were sending multiple SPs to the same healthcare provider and (b) we would be carrying out these assessments as part of a quality of care surveillance for a TB program (PPIA) being implemented by NGOs in Mumbai and Patna. In this case, the consequences of potential detection were very high, as the entire 3-year study could have been jeopardized. Further, as part of the PPIA program, providers who were part of the PPIA network were to attend trainings and workshops together. This added a risk to the SP study - for example, if providers in Mumbai and Patna were consented for an SP study, the PPIA networked providers

[3] Office for Human Research Protections (OHRP). Accessed at: http://www.hhs.gov/ohrp/policy/consentckls.html.

could discuss the identities and personal characteristics of the SPs. The combination of informed consent and congregation of providers at frequent intervention trainings (at times several are scheduled in one week) threatened the validity of our study as reported responses would not reflect the actual quality of care we were aiming to measure, while increasing the risk of SP detection.

We therefore worked closely with IRB requirements on informed consent, which is handled as per the provisions of the Government of Canada Panel on Research Ethics in the 2nd edition of the Tri-Council Policy Statement of Ethical Conduct for Research Involving Humans' Article 3.7 entitled "Alteration of Consent in Minimal Risk Research" [4]. Prior to the current study, we conducted a pilot with informed consent to validate the SP method for tuberculosis in urban India (published as Das et al. (2015) in Lancet Infectious Diseases). The results of the pilot validation study confirmed the decision to seek a waiver of informed consent in the current study. Corresponding to the requirements of Article 3.7, we documented in our pilot study that the SP approach in urban India was no more than minimal risk of participation to the SPs or providers. This was based on the following considerations:

1. Opinion data from providers in the pilot demonstrated that participation in the study did not adversely affect their practice in any way.

2. No monetary loss was incurred by the providers as the SPs, like real patients, paid the full consultation fee.

3. No added inconvenience was placed on real patients as the SPs were trained to immediately step aside if there were an emergency that demanded the doctor's attention.

4. None of the identities of the providers or their clinics were compromised since we maintained strict anonymity in the information collected and never disclosed the identity of health care providers who participated in the study.

[4] Government of Canada Panel on Research Ethics. 2012. TCPS 2 – Chapter 3, Accessed at: http://www.pre.ethics.gc.ca/eng/policy-politique/initiatives/tcps2-eptc2/chapter3-chapitre3/

5. From our observations, average consultation times in our pilot were between 3 and 7 minutes, so that would only inconvenience other patients by that time.

Based on these considerations, we requested a waiver of informed consent from the Institutional Review Board at ISERDD and the Research Ethics Board at McGill University. The requests for a waiver were reviewed and approved by both ethics committees, contingent upon the provision of a letter of full disclosure to be sent to debrief any provider who received an SP at the end of the study. The letter will offer health care providers a chance to further discuss any aspect of the findings or methodology and register any concerns; however, no individual data on any clinic or provider will be disclosed. Because our larger quality of care surveillance study has been extended to December 2019, this letter will be circulated thereafter.

S1d Provider Sampling

During the primary data collection period for this study in Mumbai and Patna, urban TB programs funded by the Bill and Melinda Gates Foundation (BMGF) and implemented by Private Provider Interface Agencies (PPIAs) in each city - World Health Partners (WHP) in Patna and PATH in Mumbai - were mapping, recruiting, and enrolling private sector providers into provider networks in both cities. At the time of sampling for our study in these two cities, we decided to stratify our provider sample by PPIA program enrollment as detailed in S7 Fig. From lane-by-lane mapping exercises conducted by the PPIA, which resulted in a universe list of private sector providers in Mumbai and Patna, we obtained lists of enrolled and not-enrolled providers in the PPIA program and merged them to produce a complete sampling universe stratified by PPIA enrollment status. We then restricted these lists based on eligibility criteria for the SP study: providers eligible for the study were those who were known to see adult outpatients with respiratory symptoms in the private health sector. The description of the program serves to support sampling weights (Table 1) applied to achieve the urban area estimates for Mumbai and Patna but stratified findings based on PPIA program enrollment are not presented in this paper.

Patna

The WHP field team conducted a street-by-street mapping exercise between January and July 2014 in urban Patna. The exercise involving mapping all health facilities and providers, as well as pharmacists and laboratories in the private health sector. Areas covered included all wards in Danapur, Phulwari Sharif and Patna blocks in Patna District. The resulting lists, along with PPIA program engagement lists and program activity data, became the raw files for the SP study sampling frames in Patna. Based on the provider qualifications captured during the WHP mapping exercise, providers were categorized by qualification (MBBS and higher, non-MBBS) for the purpose of the SP study. De-duplication of mapping entries was achievable based on the following mapping data fields: provider name, facility name, provider telephone, block, ward, and address. The non-MBBS provider list was relatively easy to clean; however, the MBBS or higher provider list was not in useful shape, which was confirmed in the field. To verify the accuracy of addresses, ISERDD and several authors of this study conducted several scoping exercises before and throughout data collection.

On December 5, 2014, BMGF and WHP initiated a second mapping of formal providers to replace the cleaned formal provider universe list, for the purpose of SP study sampling and program targeting activities. The second mapping of formal providers was particularly advantageous for the SP study for four reasons. It allowed us to (1) see what proportion of non-engaged providers were actually relevant for the PPIA program, (2) ensure that details relevant for the SP study were included in the mapping for the MBBS or higher provider evaluation, (3) ensure that good information was captured for relocating the providers for when the PPIA field team would begin sensitization, engagement, and training activities and for when the SPs were sent to them, and (4) do as much as possible to avoid any field issues, such as rare operating hours, clinics open one day a week, and sending SPs to providers not eligible for the study, such as child specialists or orthopedists.

We, the quality of tuberculosis care (QuTUB) team, worked with WHP's M&E officer and had the support of ACCESS Health International (AHI). The mapping activity was planned for ten medical officers and supervised by a field supervisor who would report to the rural and urban M&E officers. The mapping was completed at the beginning of February 2015. The mapped MBBS and higher provider universe was delivered to the QuTUB team in two parts, since the SP field team was waiting for an updated schedule of interactions. Because the updated mapping universe was delivered in two parts, the sampling for non-engaged MBBS and higher providers was broken into two strata: MBBS and higher providers mapped between December 5 and January 9, and MBBS and higher providers mapped from January 10 to beginning of February. There were no significant differences between these two groups (results available upon request).

At the end of both mappings, the cleaned universe in Patna consisted of 1,841 formal providers and 1,338 informal providers (see **S7 Fig**). Patna SP interactions occurred between 21 November 2014 and 28 February 2015. SP interactions were done first for informal (non-MBBS) providers, followed by formal (MBBS or higher) providers.

Mumbai

Between January and June 2014, PATH contracted out street-by-street mapping activities in Mumbai of private health facilities, originally planned for 12 high TB burden wards and 3 high-slum population wards, which account for 86% of the Mumbai slum population and 70% of the Mumbai population. The mapping activity was done by two community-based organizations (Alert India and MJK). Mapping was initiated in G-North (high TB burden), M-East (Chembu slums and presence of Chest Physicians), P-North, and S wards. By June 2014, the exercise resulted in a provider universe of 8897 locations in 18 wards, which were 12 fully mapped wards, partial mapping of H-East, P-South, and R-South wards, and minimal mapping in the remaining wards. Of the 8897, 2804 were chemists, 3591 were AYUSH

practitioners (BAMS, BUMS, BHMS, DAMS, DHMS, LCEH), 671 less than fully qualified allopathic providers, 1290 MBBS, 463 MDs, and 78 chest physicians. Similar to Patna, a provider universe list produced by the IMS Health company was reviewed, but a decision within the PATH team and separately within the QuTUB team was made not to use or append this list.

Since the network established by PATH aimed to move with the providers regardless of where and in how many facilities they practiced, the final QuTUB sampling frames were the result of thorough cleaning, scoping, and verification exercises in collaboration with the PATH team. Before fieldwork began, an individual (R) hired by ISERDD together with a representative from AHI (NC), both from Mumbai, heavily scoped the field to check for addresses and to gather additional information that would be useful before the ISERDD field team moved to the city. The information that was gathered included, but was not limited to: identifying potential SP recruits, looking at the transportation systems, reporting the setup of clinics and health facilities, collecting GPS locations of providers, and correcting provider and facility names while capturing consulting fees, first points of contact in a facility, and outpatient department (OPD) timings.

Four wards out of the 15 original focus wards of the PPIA program were selected for the majority of Mumbai SP surveillance to minimize geographical spread of the SP study. This was because increasing the geographic scope of the city would increase logistical difficulties as presented by Mumbai's transit system and scale. In mid-January 2015, a meeting was conducted between the PATH and QuTUB teams to agree on the four study wards. After assessing upcoming PPIA program efforts, access to transportation, mixture of unregistered and registered slums, and available data on the wards (e.g., total ward population, slum population, and proportion of slum population figures as per the 2011 census), F-North, K-East, L, and P-North were selected. Relative to other mapped wards, these four wards have a higher slum population (with the exception of F-North), and they also have more providers and more networked providers. Together, these wards were a good representation of

areas for PPIA Mumbai efforts. As noted in sections below, these wards were used for constructing the AYUSH (see **S7 Fig**) and non-PPIA facility ("hub") samples.

In addition to PPIA hubs, there were non-networked private hospitals and single provider private clinics that met the PPIA hub criteria (i.e., in-house or nearby digital X-ray, pharmacy, and chest physician) and could provide a reasonable comparative 'apples-to-apples' estimate of the quality of major facilities not enrolled in the program. The list of non-PPIA hubs was created with the assistance of PATH, who verified whether each one would be a comparable candidate for enrollment into the program. The list consisted of health facilities that met the criteria for the PPIA network and located in the four study wards: F-North, K-East, L, and P-North wards. An initial list of eligible but non-PPIA 'hubs' was created by PATH in January 2015, and this list was revisited and updated in April 2015. The process of updating the list at the end of April 2015 involved PATH team members and FOs going through the list and removing any facilities that had been networked since July 2015, when SP interactions began for the eligible, yet non-PPIA hubs.

The FOs also went through the full universe list again to make sure the PPIA networking criteria still applied, and any duplicates were removed (at least 12 sets of duplicates or triplicates). The final list was frozen on April 28, 2015, and the QuTUB team flagged facilities that were ineligible for the SP study (i.e., children hospitals) before establishing the sampling frame for non-PPIA hubs. The final list contained 78 eligible, yet non-PPIA hubs, and 11 were excluded from actual SP visits, since they were children or orthopedic hospitals and the SP cases did not reflect pediatric TB or extrapulmonary TB (see **S7 Fig**).

Furthermore, we ascertained how PPIA providers (MBBS and higher) practice at PPIA hubs and non-PPIA locations. To expand on this, it was common for individual PPIA providers practicing at PPIA hubs to also have other private practices, which we deem as 'non-PPIA locations' or 'non-networked locations'. These are locations where the providers were confirmed to not be networked regardless of being networked in the PPIA at a different location. To confirm the PPIA status

of these private practices, an initial mapping with verification activities for these 'non-networked locations' occurred between April and July 2015. For initial mapping, two PATH team members in collaboration with the QuTUB team procured a list of practices for 150 PPIA providers, who were MBBS, MDs, and MD/Chest Physicians and who were all networked at known PPIA hubs (see **S7 Fig**). Of the 150, 136 met the inclusion criteria (i.e., providers who see adult pulmonary patients) for the SP study.

In terms of the process, FOs conducted the non-networked location mapping in two phases. The first phase, which was circulated to the QuTUB team on July 3rd, contained 153 locations for 62 of the 136 eligible providers and was collected from FOs in R South, N, G North, M East, M West, and F North wards (providers were interviewed at their networked location linked to a ward and a corresponding FO). This resulted in a list of non-networked locations for 62 providers where: 18 providers had 1 location; 21 providers had 2 locations; 10 had 3; 6 had 4; 4 had 5; 2 had 6; and 1 had 7. PATH team members reviewed these locations to determine their networked statuses, and AHI and ISERDD teams conducted scoping for eligibility into the SP study (e.g., government facilities were ineligible for the SP study). The second phase contained details for the remaining 74 providers. For the verification activities, ISERDD and AHI (NC) did on-ground scoping to double-check for any government facilities, ensure there were no duplicates, confirm outpatient hours of the providers, and check whether providers had on-call duties at any of the locations. At the time of verification, ISERDD had already completed all PPIA and non-PPIA hub walk-ins, and favorably, the team was also able to map whether or not any of these non-networked locations had already been visited as a non-PPIA hub. If so, facility IDs matching with those non-PPIA hubs were mapped back to the sample and analytically used also for 'non-networked' interactions.

S1e Assignment of SP Cases to Providers

Patna

Two different samples were randomly generated for non-MBBS providers:

1. Uniform-probability random sample of non-PPIA, non-MBBS providers (with reserves)

2. Uniform-probability random sample of PPIA, non-MBBS providers (with reserves)

Drawn from the full provider universe of all wards in Danapur, Phulwari Sharif, and Patna blocks of urban Patna (source: WHP mapping data), the random samples for the PPIA and non-PPIA samples for non-MBBS providers were restricted to the following geographical space: 40/40 wards of Danapur block, 28/28 wards of Phulwari Sharif block, and 34/73 wards. These wards were purposively selected areas of Patna block and decided in collaboration with WHP to cover areas which could have higher TB burdens - Kankarbagh, Rajendra Nagar, Patna City, and Patliputra Housing Colony neighborhoods. PPIA engagement status used for sampling was frozen on September 25, 2015.

MBBS and higher providers were also selected with stratified uniform-probability random sampling. Engagement status and program activity was frozen on September 25, 2014. The two samples were:

1. Uniform-probability random sample of non-PPIA, MBBS and higher providers

2. Uniform-probability random sample of PPIA-engaged, MBBS and higher providers

Similar to the non-MBBS geographical sampling, the random samples for MBBS providers were drawn from the same geographical areas as the random sample for non-MBBS providers. One field challenge surmounted in December 2014 for the non-PPIA, MBBS sample, which resulted in halting the fieldwork in December 2014. A quarter of the interactions attempted resulted in specialists irrelevant and ineligible for the study, and others had closed shop and moved since the WHP mapping exercise. Since WHP initiated a new mapping activity for MBBS and higher providers in the same month, as described in the mapping section, we took advantage of this

to resample this group, as well as to conduct a pre-screening selection for an impact evaluation. Since the re-mapping and SP fieldwork coincided, we re-sampled the non-PPIA, MBBS and higher group in two waves as the mapping data arrived: stratum 1 and 2. Replacing the non-PPIA, MBBS and higher sample, Stratum 1 contained 150 providers and Stratum 2 contained 100 providers. A total of 500 total assigned interactions were attempted, of which 442 were completed. There were no significant differences between the two strata (available upon request).

Additionally, on January 7, 2015, 120 MBBS and non-MBBS providers who had already received an SP portraying Case 1 were scheduled another Case 1 portrayed by a different SP actor. A four-week grace period at minimum between the first visit and the beginning of the revisit interaction period was designed to minimize detection. The objective of this was to see how consistent providers were with patients of identical case presentation. Case 1 revisits were scheduled after the first non-engaged, MBBS provider schedule was halted and before the non-engaged MBBS sample could be replaced from WHP remapping. This meant that a subset of non-MBBS and MBBS providers received a second Case 1, regardless of PPIA or non-PPIA engagement status and regardless of sampling strategy. For the Case 1 revisits, ISERDD used the full cohort of 12 SPs while finishing up any remaining interactions left over from the previous informal and engaged formal samples.

Mumbai

Non-MBBS sampling (see **S7 Fig**) was restricted AYUSH practitioners and to four study wards as agreed with PATH. Engagement statuses of AYUSH were frozen on January 24, 2015. There were three samples that resulted:

1. Uniform-probability random sample of non-PPIA AYUSH in K-East and L wards
2. Uniform-probability random sample of non-PPIA AYUSH in F-North and P-North wards

3. Census of PPIA AYUSH in all four wards

Schedules were given to ISERDD in two groups: AYUSH in K-East and L wards in one group, and AYUSH in F-North and P-North wards in a second group. For both groups, reserves were provided for the non-PPIA practitioners based on ward. All AYUSH interactions were randomly assigned to be conducted either in the morning or evening hours.

Next, general walk-ins without appointments to hubs were conducted by having the SP enter the health facility and go to the doctor suggested by the receptionist or intake nurse, without regard for PPIA status of the provider, at both PPIA-registered facilities and non-PPIA facilities. There were two samples:

1. Non-PPIA hub walk-ins at purposively selected locations in the four wards
2. PPIA hubs walk-ins (census of all PPIA hubs) across 15 wards

All hub walk-ins were conducted during specific times that were given to the field team. For PPIA hubs specifically, walk-ins were conducted during hours any networked doctor were scheduled to practice, unless an appointment was scheduled by the receptionist or intake nurse. If, during the consultation, the SP was told to go to another doctor at that moment, the SP was instructed to do so and any aspect of the interaction was also recorded on the same form. On May 1, 2015, before sending SPs to the field, ISERDD supervisors met with the PATH team to go over all the PPIA hubs, their locations, the facility layout, and the networked providers.

Walk-ins were first conducted among 100 PPIA hubs. Only Case 1 interactions were conducted. Once PPIA hub walk-ins were completed, walk-ins were done at non-PPIA hubs. Since we had to still confirm whether these facilities would accept a presumptive TB walk-in patient, all non-PPIA hubs were sent an SP portraying Case 1. Only after Case 1 interactions were successfully completed, we randomly assigned half to receive Case 2 and then randomly assigned another half to receive either Case 3 or Case 4. None of the SPs trained as Case 4 carried a sputum report to the non-PPIA hubs. To avoid any risk of the same SP running into the same

provider across locations, the following field protocols were put in place: (1) SPs were to remain cognizant and maintain a list of providers they had visited, and (2) supervisors were to review a provider directory when assigning SPs for interactions. At least two PPIA providers were seen by SP1 during the non-PPIA walk-in, while 54 of 93 PPIA facility walk-ins resulted in the SP seeing a PPIA provider.

We then targeted additional PPIA providers at facilities where they practiced as part of the PPIA (see **S7 Fig**). Updated lists for providers, mostly MDs and MD Chest Physicians, networked by the PPIA were procured in January 2015 and then again in April 2015. A total of 136 providers at PPIA hubs were eligible for the SP study (ineligible providers, such as pediatricians and orthopedist specialists, were excluded) across 100 health facilities, resulting in 98 successful interactions. When scheduling the providers and locations that would receive SPs, our top priority was to maximize the total number of interactions we could conduct while reducing risk of detection. In order to do this, we had to work around three issues: (1) providers who were networked at multiple locations, (2) PPIA hubs that had more than 3 networked providers, and (3) providers selected who had already been seen during the walk-in visits. Selecting the eligible providers was done manually and checked several times.

Among the 136 providers, we were aware of 19 who were networked at multiple locations (13 providers at 2 facilities, 2 at 3, 4 at 4 = 48). To maximize the total number of interactions, we then selected these 19 providers at the facility that had the fewest number of other practicing doctors. Among the PPIA hubs, there were six hubs considered as large, defined as having more than 3 networked providers (2 hubs with 5 providers, 1 with 7, 1 with 9, 1 with 10, 1 with 11 = 57). To avoid detection, we decided that no PPIA hub would receive SPs for more than three providers. In order to select the three providers at the large hubs, we attributed the eligible networked doctors to the number of TB cases they had reported to the PPIA program and ranked them from highest to lowest. We then removed all the providers located at multiple locations since we had already selected another one of

their networked locations. Three providers were then selected by taking the highest notifying, the lowest notifying, and a random provider who had not been selected yet and who could be either notifying or not to the program.

All PPIA providers at PPIA hubs received SPs portraying Case 1 (however, if SPs as Case 1 had already seen the networked doctor during a walk-in, we did not assign another Case 1 to this doctor, and the assumption was the walk-in observation for Case 1 could be used for the provider targeted interaction for Case 1). A random half received Case 2. Another random half received Case 3 with a sputum report in addition to Case 4 without a sputum report, with the other half was assigned to not receive Case 3 and to receive Case 4 with a sputum report. To minimize detection risk, the field team conducted interactions at the six large facilities last.

Finally, we specifically targeted PPIA providers at their other practice locations where they were not considered part of the PPIA (non-networked locations or "NNLs"). From the verified list of non-networked locations, NNLs that were government facilities and outside Mumbai city were excluded for sampling. Some of the interactions, though not deliberately scheduled for ISERDD, took place during the walk-ins at non-PPIA locations when providers who also practiced within the network were encountered by chance. Scheduling for ISERDD was done in 4 waves as the mapping was done in parallel to fieldwork, and verification process was also done with SP1 case.

PPIA providers at NNLs were scheduled to receive Case 1 before any other cases. This was done to serve as a final verification for location eligibility. Case 1 was randomly assigned to one NNL for the providers who SPs visited in the previous sample at a networked location. If the provider was known to have multiple NNLs, we gave preference to the location where he or she was not on-call or working at an actual large PPIA hub before random selection, since large PPIA hubs had already received at least 3 provider-specific SP1s. All on-call locations and other NNLs for each provider was made available as a reserve list to the field team. All PPIA providers at their NNLs were assigned Case 1. The half that did not receive Case

2 at the networked location were assigned to receive them at the NNL. The half that received Case 4 with a sputum report were assigned to receive Case 4 without a sputum report.

References

1. Alderman H, Das J, Rao V. Conducting ethical economic research: complications from the field. In: DeMartino G, McCloskey D, editors. The Oxford Handbook of Professional Economic Ethics. Published online; 2014. Accessed at: http://www.oxfordhandbooks.com/view/10.1093/oxfordhb/9780199766635.001.0001/oxfordhb-9780199766635-e-018.

2. Das J, Kwan A, Daniels B, Satyanarayana S, Subbaraman R, Bergkvist S, et al. Use of standardised patients to assess quality of tuberculosis care: a pilot, cross-sectional study. The Lancet Infectious Diseases. 2015;15(11):1305–13. doi: 10.1016/S1473-3099(15)00077-8. PubMed PMID: 26268690.

3. Satyanarayana S, Kwan A, Daniels B, Subbaraman R, McDowell A, Bergkvist S, et al. Use of standardised patients to assess antibiotic dispensing for tuberculosis by pharmacies in urban India: a cross-sectional study. The Lancet Infectious Diseases. 2016;16(11):1261-8.
