# Supplementary material for: Variations in the quality of tuberculosis care in urban India: A cross-sectional, standardized patient study in two cities
Source: PLoS Med. 2018 Sep 25;15(9):e1002653. doi: 10.1371/journal.pmed.1002653 (PMC6155454; doi:10.1371/journal.pmed.1002653)
Supplement: S2 Text — (PDF) [file pmed.1002653.s002.pdf]

S2 Text: Statistical Methods

S2a Weighting

Based on our sampling strategy in **S1 Text**, our city-level estimates of the quality of care in Mumbai and Patna extrapolate from the sampling frame to the full population of private health care providers in each city. To calculate averages and differences within and across cities, we utilize inverse probability weights to satisfy the following:

1. Each city-case combination (Patna Case 1, Mumbai Case 2, etc) has a total sum of weights equal to one. Therefore each case is equally weighted within each city, and the two cities have equal total weights.

2. Within each city-case combination, the sum of weights for (A) MBBS-qualified and above and (B) non-MBBS-qualified providers is exactly equal to each group's prevalence in the city as a whole.

3. Within each city-case-qualification group, the relative total weights for (A) PPIA and (B) non-PPIA providers are exactly proportional to each group's prevalence in that city and qualification stratum.

By satisfying these conditions, the weight on each interaction is calculated such that our estimates take the values that they would if we had sampled exactly at random from the city as a whole, assuming that our sample is representative of that provider mix. There are 32 weighting groups: one for each city, case, qualification, and PPIA status ($2*4*2*2$). Under the assumption that the providers we sampled from our sampling frames are representative of similar providers throughout the city, the resulting estimates are representative of the choice of a random provider within the city for each case presentation. When we report statistics of the form "X of Y interactions (N%)", Y is the whole number of interactions observed, X is the whole number of interactions in which the outcome occurred, and N is the population-level estimate calculated using the weights detailed above.
