# Supplementary material for: Variations in the quality of tuberculosis care in urban India: A cross-sectional, standardized patient study in two cities
Source: PLoS Med. 2018 Sep 25;15(9):e1002653. doi: 10.1371/journal.pmed.1002653 (PMC6155454; doi:10.1371/journal.pmed.1002653)
Supplement: S3 Text — Assessing variation in provider management of cases; ANOVA decomposition of explained variance; consistency of individual providers across visits; distributions of providers within city-qualification groups. (PDF) [file pmed.1002653.s003.pdf]

S3 Text: Supplementary Results

S3a Assessing variation in provider management of cases

One potential explanation of the low observed rates of correct management in the overall provider sample is that our methodology is limited by the fact that standardized patients were only assigned to complete a "first and one-time visit" for each case with the provider, and no follow-up visits were made in any case. One possibility, then, is that most providers follow some alternate "standard" treatment regimen for chest symptomatic cases on the first visit which is appropriate for a high local prevalence of self-limiting diseases, after which the provider considers the possibility of more serious conditions, such as TB, on follow-up visits.

To assess this hypothesis, we examine all Case 1 visits in which correct management for TB was *not* provided. If this is a viable explanation, we would find that, for example, a large number of providers give similar regimens focused on alleviating symptoms (such as cough syrups) and that would allow minor respiratory conditions – particularly viral ones for which there is no effective direct treatment – to run their natural course. **Fig 2** details these results as a Venn diagram of major medication classes given to Case 1 SPs when the appropriate TB treatment was not provided.

Rather than observing one or two major alternative management regimes for the naive TB Case 1 scenario, we observe a broad range of combinations of four primary drug medication classes – cough syrups, broad-spectrum antibiotics, fluoroquinolone antibiotics, and steroids – as well as a large group of providers who follow a completely different course of treatment. This suggests that there is not a single consistent alternate treatment being applied on initial visits, and therefore we have little evidence to suggest, for example, that these providers will quickly converge on a suspicion of TB in follow-up visits; field work is ongoing in this area.

While the data is richest for Case 1 since such an interaction was completed with nearly every provider, we find that management choices vary widely across every case scenario, as illustrated in **S1 Fig**, which breaks each case down into

(1) no correct management and (2) correct management, each divided into (A) no additional medications and (B) additional unnecessary medications. Since the provider mix used for estimation of these proportions is held constant across cases as a result of our weighting approach, this result can be understood as a rough estimate of the effect of varying the information available to the provider on that first visit by changing the case presentation. Every case receives all types of treatment outcomes, and in all cases the predominant treatment outcome includes some form of unnecessary medication, suggesting wide variation in treatment regimes in response to each of our scenarios.

Similarly, providers had a wide variety of behaviors when requesting follow-up visits from SPs (which the SPs recorded but did not complete), an outcome which was not pre-specified. **S2 Fig** shows these requests in detail, broken down by reason. In most cases, follow-up visits were specifically requested for test results; however, when the provider did not treat the case correctly but also gave no medication, typically the follow-up request was for a set number of days or if the condition did not improve on its own. Field work is ongoing that examines the evolution of provider behavior if the patient were to return as requested.

S3b ANOVA decomposition of explained variance

We test one major hypothesis that could explain this wide diversity in treatment approaches. Using an ANOVA decomposition, we investigate whether these diverse treatment choices are well-predicted by observable provider characteristics: that is, whether Patna MBBS providers are simply systematically different in practice from, say Mumbai non-MBBS providers and that the diversity of treatment approaches can be explained in this way. To do this, we use an ANOVA decomposition to calculate the relative contributions of each of the major provider characteristics used in sampling (except PPIA status) to quality outcomes. We calculate the R-squared of a series of regressions including the three primary characteristics: city, case, and qualification; to maximize the potential explained variance, we then include fixed

effects for each individual standardized patient and for an interaction model that allows the coefficients to vary across city and case. **S3 Fig** illustrates these results in graphical form.

For all characteristics, explained variance across the various potential management behaviors is relatively low. Even with all characteristics combined, the total explained variance is less than 25 percent for every outcome except the chest X-ray (with the most predictive power provided by provider degree qualification) and correct management (with the most predictive power provided by the case scenario). This result emphasizes that while providers respond to the case presentation, and that the two qualification groups differ systematically in their decision whether to utilize diagnostic testing like the popular chest X-ray, accounting for all our macro-level characteristics leaves a large amount of unexplained variance in our representative sample. In other words, there is a large amount of variation remaining across providers even within our primary stratification on observable characteristics.

S3c Consistency of individual providers across visits

In a subsample of 109 providers in Patna, we sent a second Case 1 visit portrayed by a different individual SP, to assess the degree to which the cross-provider variations documented above occurred *within* providers as well. In doing so, we seek to understand whether individual providers have a high degree of uncertainty about what to do when faced with a particular scenario – and therefore vary their practice widely even when confronted later with a similar patient; or whether individual providers are fairly "sure" about the course of action they took in a particular case and are therefore likely to repeat it.

If there is diversity in management approach within individual providers, the key route to increasing care quality would be to improve the average quality or reduce the variance of those providers through some sort of education or training program. On the other hand, if providers demonstrate very stable management decisions in reality, then improvements in quality for patients can be achieved through allocative

efficiency gains – that is, redirecting patients to consistently high-quality providers who are at efficient prices relative to the market as a whole.

In this micro-experiment, for each management behavior, we estimate the proportion of re-visited providers who took the same action in both of their Case 1 interactions. For example, we calculate the proportion of providers who either correctly managed Case 1 in *both or neither* of the two interactions. **S4 Fig** illustrates these results for each of our primary outcome measures.

Unlike the high degree of cross-provider variation we highlighted so far, this subsample suggests that each individual provider displays a high degree of stability over time in response to the same case scenario, a phenomenon we call "idiosyncratic consistency". In this hypothesis, each individual provider has a stable set of practices they use in response to a given case presentation, but that practice is not widely shared across other providers that share the same set of location and qualification characteristics.

S3d Distributions of providers within city-qualification groups

In this section we highlight the extent to which there is variation in quality beyond the observable and binary measures we focus on in the main text. Here, we use a proxy measure of each provider's underlying quality, which has been shown to be highly correlated with correct management. We calculate the proportion of checklist questions that each provider asked of the Case 1 SP. Since these are continuous percentages, we can investigate the degree of variation remaining in the provider distributions after separating them by city and qualification. **S5 Fig** illustrates these distributions as kernel densities (smoothed histograms) separately for MBBS and non-MBBS providers in the two cities, with relative frequencies on the vertical axis.

There are several interesting characteristics of these distributions. First, the mean of MBBS providers is significantly higher than those of non-MBBS providers within each city. Second, consistent with the variation documented across settings,

the non-MBBS providers are significantly better in Mumbai than in Patna for Case 1, and MBBS providers in Mumbai are on average of higher quality than the mean MBBS in Patna. Beyond the differences in means, there is substantial variation in quality within each qualification. In both cities, non-MBBS providers display a right-skewed distribution, with most providers concentrated at the lower part of the quality distribution. Unlike non-MBBS providers in Patna, those in Mumbai have a longer "tail", with significant overlap between the non-MBBS and the MBBS providers in the city. On the MBBS side in both cities, there is a very broad range of providers. MBBS always contribute the top performers, but in both cities there are MBBS who perform less checklist questioning than a large portion of the city's non-MBBS providers.

Finally, we provide an alternate breakdown of non-MBBS providers in Patna, where both AYUSH and non-AYUSH providers practice. In **S6 Fig**, we show logistic regressions between the two types of providers. Those with little or no formal qualification were more likely to manage cases correctly, largely via referral, and also gave unnecessary medications of all classes more frequently.
