# Supplementary material for: Variations in the quality of tuberculosis care in urban India: A cross-sectional, standardized patient study in two cities
Source: PLoS Med. 2018 Sep 25;15(9):e1002653. doi: 10.1371/journal.pmed.1002653 (PMC6155454; doi:10.1371/journal.pmed.1002653)
Supplement: S4 Text — Study design and methods, and data analysis plan. (PDF) [file pmed.1002653.s004.pdf]

S4 Text: Pre-Analysis Plan from Study Protocol

Excerpt from:

Revised Research Protocol
Reviewed by McGill University Biomedical B Research Ethics Board
REB No. 13-269-BMB
Version date: 18 July 2014

**Project Title:**	Quality of Tuberculosis Care in Urban India
**Lead Investigators:**	Madhukar Pai, MD, PhD<br>*Associate Professor, McGill University &*<br>*Associate Director, McGill International TB Centre*<br>Jishnu Das, PhD<br>*Lead Economist, World Bank &*<br>*Centre for Policy Research, New Delhi*
**Partner Institute:**	Institute of Socio-Economic Research on Development and Democracy (ISERDD), New Delhi
**Study Sponsors:**	Bill & Melinda Gates Foundation
**Pilot Study Sponsor:**	Grand Challenges Canada

S4a Study design and methods

Step 1: In this step, the list frame for sampling providers will be obtained from the PPIA agencies in Mumbai and Patna, stratified by whether providers are engaged with PPIAs or not. The final components of this step are (A) finalizing the localities in each city where the study will be conducted and (B) finalizing the list frame with basic details and locations (via GPS coordinates) of each provider, along with contact details. This step will be completed jointly between the PPIAs and the PIs.

Step 2: The second step is the field work. The main project survey using SPs (and vignettes, on a sample of providers) will be conducted over the year with 6-monthly reports submitted on the findings using pre-defined formats from the pilot project. These 6-month reports will be submitted to PPIAs as feedback on how they can improve their performance and focus on areas where providers are found deficient (without identifying any provider by name or exact location). We have also developed an integrated platform for data entry and production of "on-demand" reports. This platform is pre-programmed, so that every time an SP

interaction is successfully completed, the reports are updated and the information is instantaneously available. The format of the report is shown in the Additional Documentation.

Step 3: The survey will be repeated in year 2 using the same approach as Step 2. The final step is the analysis of the change between baseline and endline surveys, and publication of research findings.

S4b Data analysis plan

For all cases, providers will be assessed on 3 dimensions: the fraction of essential checklist history and examinations or tests completed/ordered, relative to the standards described in the Standards for TB Care in India, the accurate diagnosis of the case, and the treatment that they prescribe, as compared to the expected standard of care (again, using Indian Standards of TB care as the benchmark).

Main outcomes include average visit length; essential history questions asked; correct diagnosis given; essential TB investigations ordered; drugs dispensed or prescribed (focusing on antibiotics, steroids, and quinolones, because of potentially harmful effects); appropriateness of TB regimens prescribed; amount of money paid by SP.

The data reporting formats (see Additional Documentation) show the key variables we will be including for each SP case. Our approach to coding of medicines/pills is also presented in the Additional Documentation. Coding of prescriptions will be done by two members of our expert panel of physicians. Unlabeled pills will be coded as unlabeled and not analyzed further. The following will be our main stratification variables in the main project:

1. Public versus private versus dual practice

2. MBBS versus less than fully qualified

3. Offering PPIA services versus not

In addition, we will be able to assess differences in quality of care provided to male versus female SPs, but this would be a secondary analysis.

Based on our pilot study in Delhi, we will measure the quality of providers' medical care by measuring adherence to TB-specific checklists of essential and recommended care, the likelihood of correct diagnosis, and the appropriateness of treatment. Essential care refers to questions providers must ask and examinations providers must conduct to accomplish a basic diagnosis of TB or protect a patient from serious harm, as determined by Indian Standards for TB Care and International Standards of TB Care, Version 3.

Recommended care refers to essential care plus other required questions and examinations so that providers can develop a reasonable differential diagnosis — for instance, to distinguish between bacterial pneumonia and TB in short duration cough/fever presentation. To collect each of these measures, SPs will be debriefed with a structured questionnaire (see Additional Documentation) within one hour of the interaction with the healthcare provider. Medicines and prescriptions dispensed in the clinic will be saved (and photographed), and the medicine names will be recorded when a label is present. The SP method will permit estimates of case detection rates since TB is pre-specified in the SP design (while vignettes cannot offer this). Our methodology will also allow for valid quality comparisons across different types of doctors and clinics, and allow comparisons over time.

Stata 12 software will be used to compute unadjusted means and standard-errors of outcome variables and to calculate regression-adjusted means and robust standard errors. Regression will be performed to generate coefficients of three separate regressions of provider and clinic characteristics (an indicator for working in the private sector, an indicator for having no medical qualifications, an indicator for whether a provider is in the PPIA network or not) on the percentage of recommended questions asked and exams performed, the relative risk of giving any diagnosis, and the relative risk of giving a correct treatment.
