# Supplementary figures and images for: Variations in the quality of tuberculosis care in urban India: A cross-sectional, standardized patient study in two cities
Source: PLoS Med. 2018 Sep 25;15(9):e1002653. doi: 10.1371/journal.pmed.1002653 (PMC6155454; doi:10.1371/journal.pmed.1002653)

S1 Fig: Correct management of SP Case scenarios, with alternate definitions

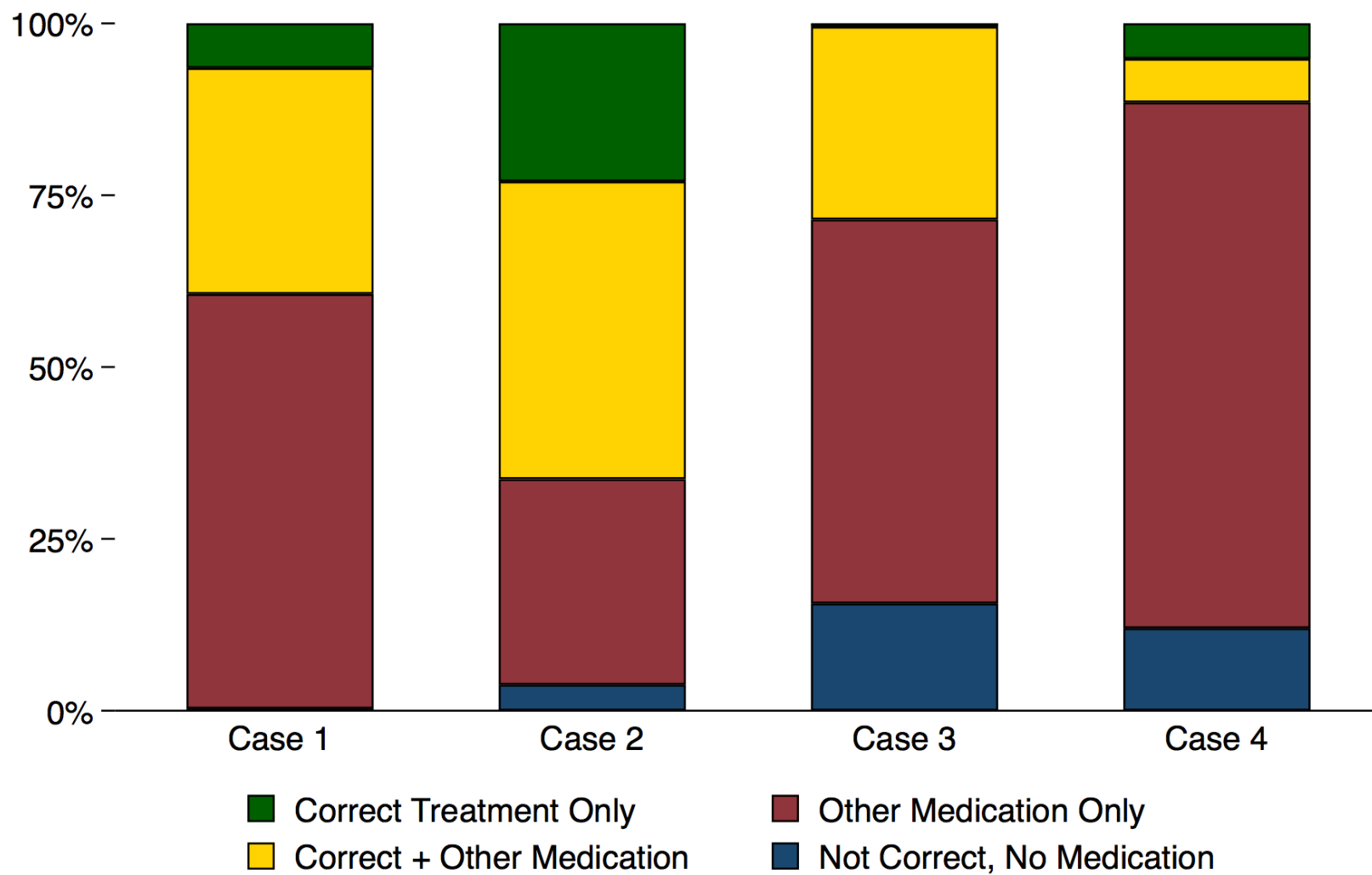

Supplement: S1 Fig — SP, standardized patient. (PDF) [file pmed.1002653.s005.pdf]

S3 Fig: ANOVA decomposition of quality correlates

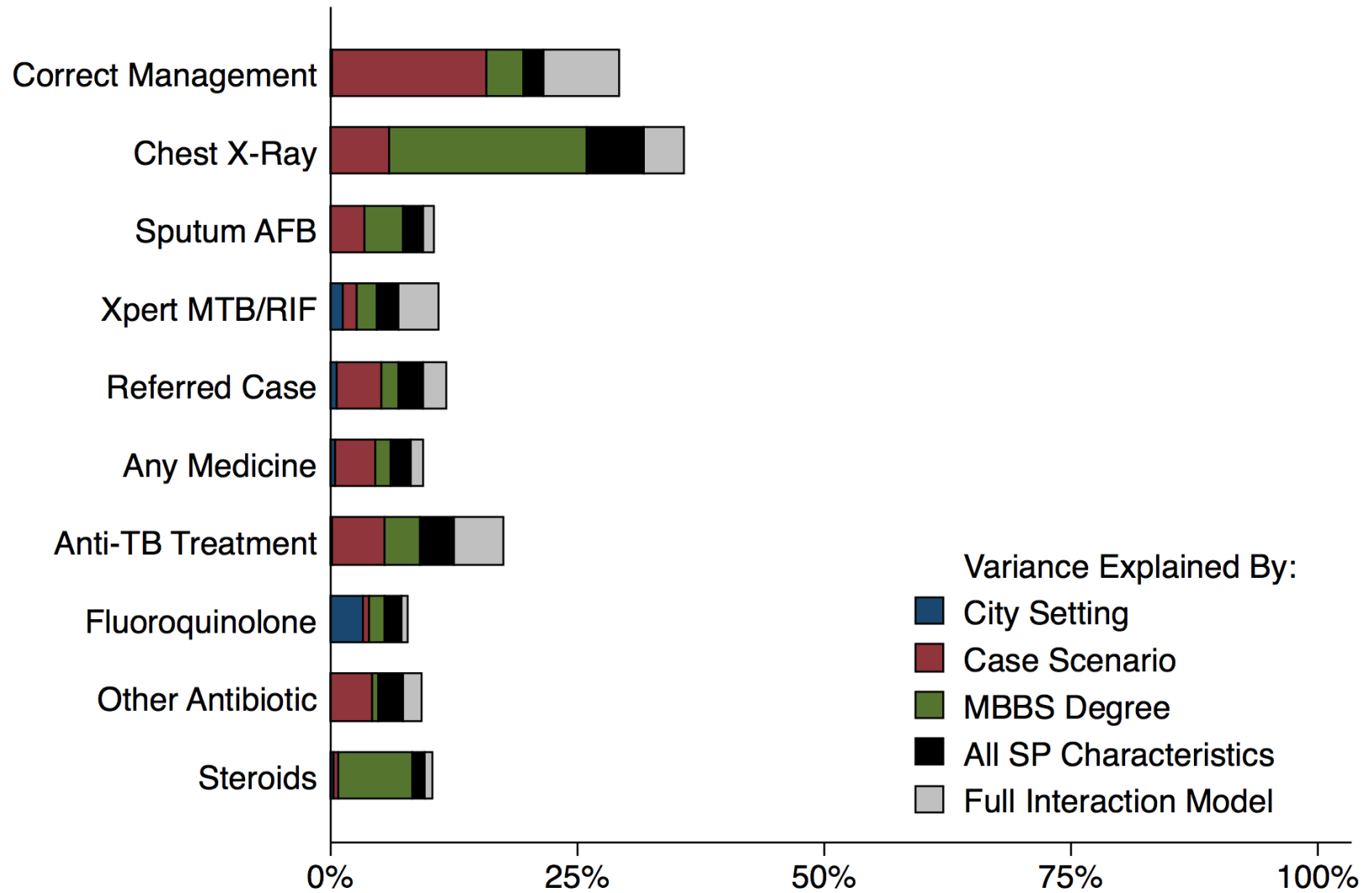

Supplement: S3 Fig — (PDF) [file pmed.1002653.s007.pdf]

S4 Fig: Consistency of providers in repeated Case 1 visits

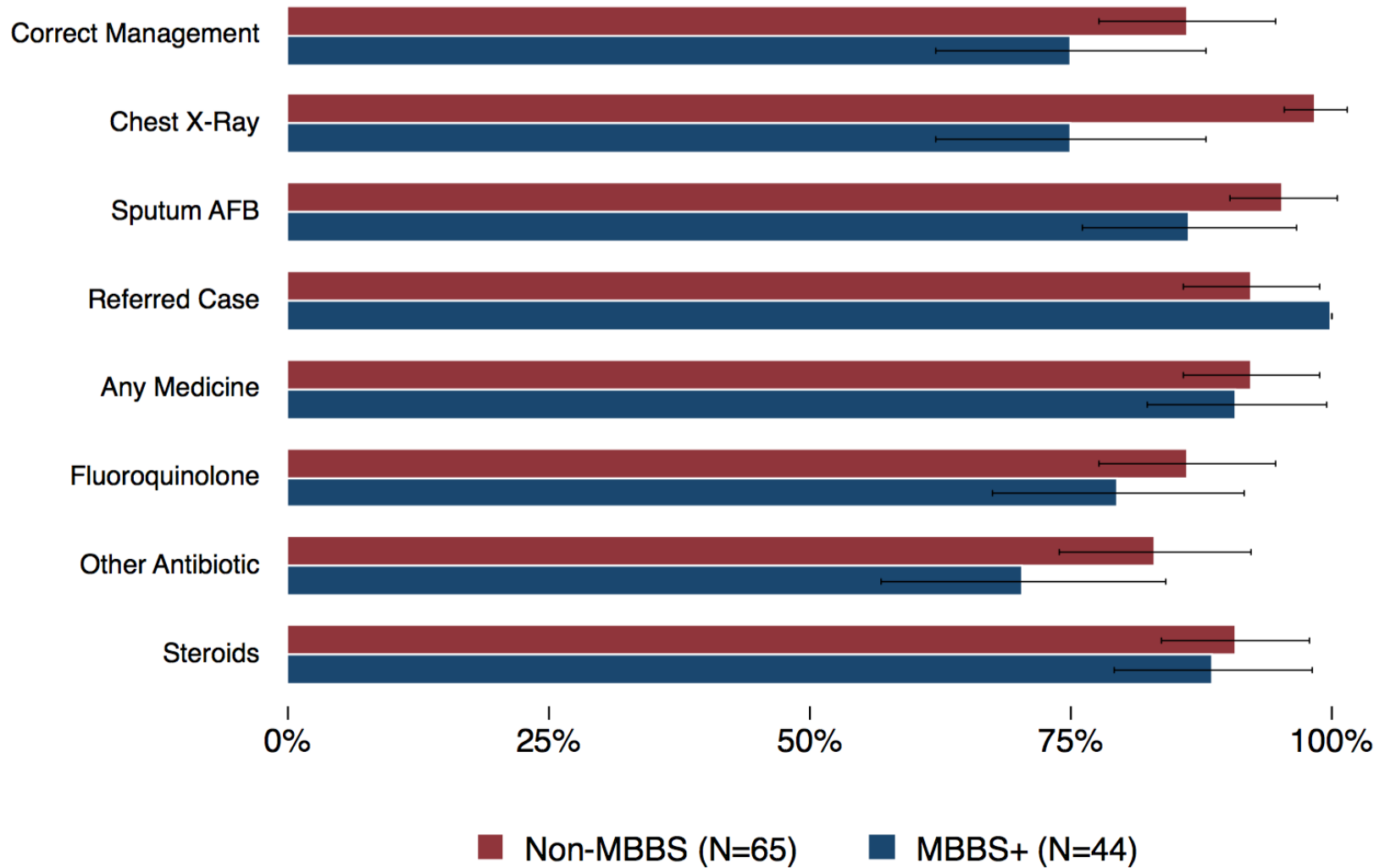

Supplement: S4 Fig — (PDF) [file pmed.1002653.s008.pdf]

S5 Fig: Distributions of checklist completion in Case 1 visits

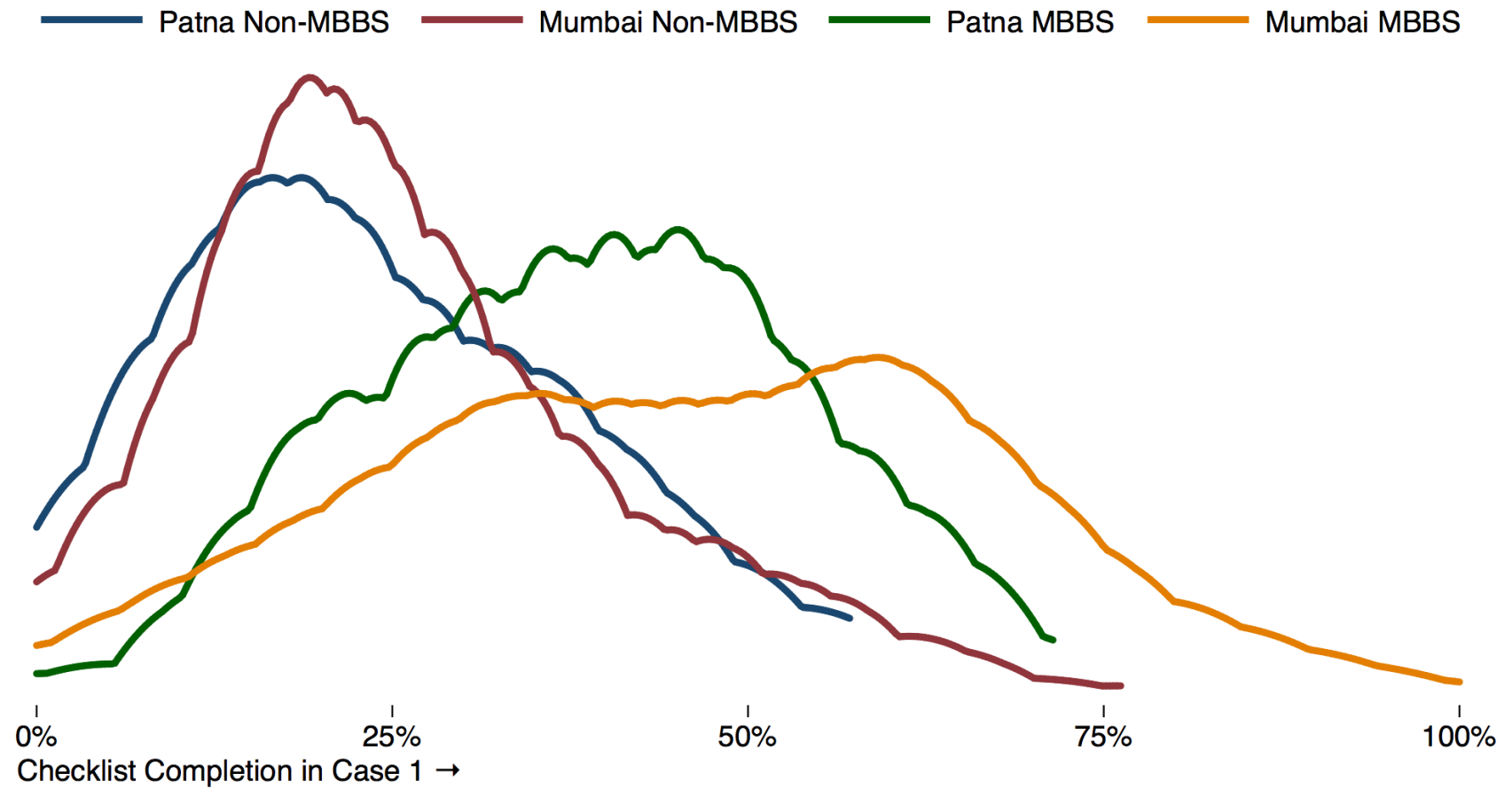

Supplement: S5 Fig — (PDF) [file pmed.1002653.s009.pdf]

**S6 Fig: Comparison between AYUSH and non-AYUSH treatment outcomes**

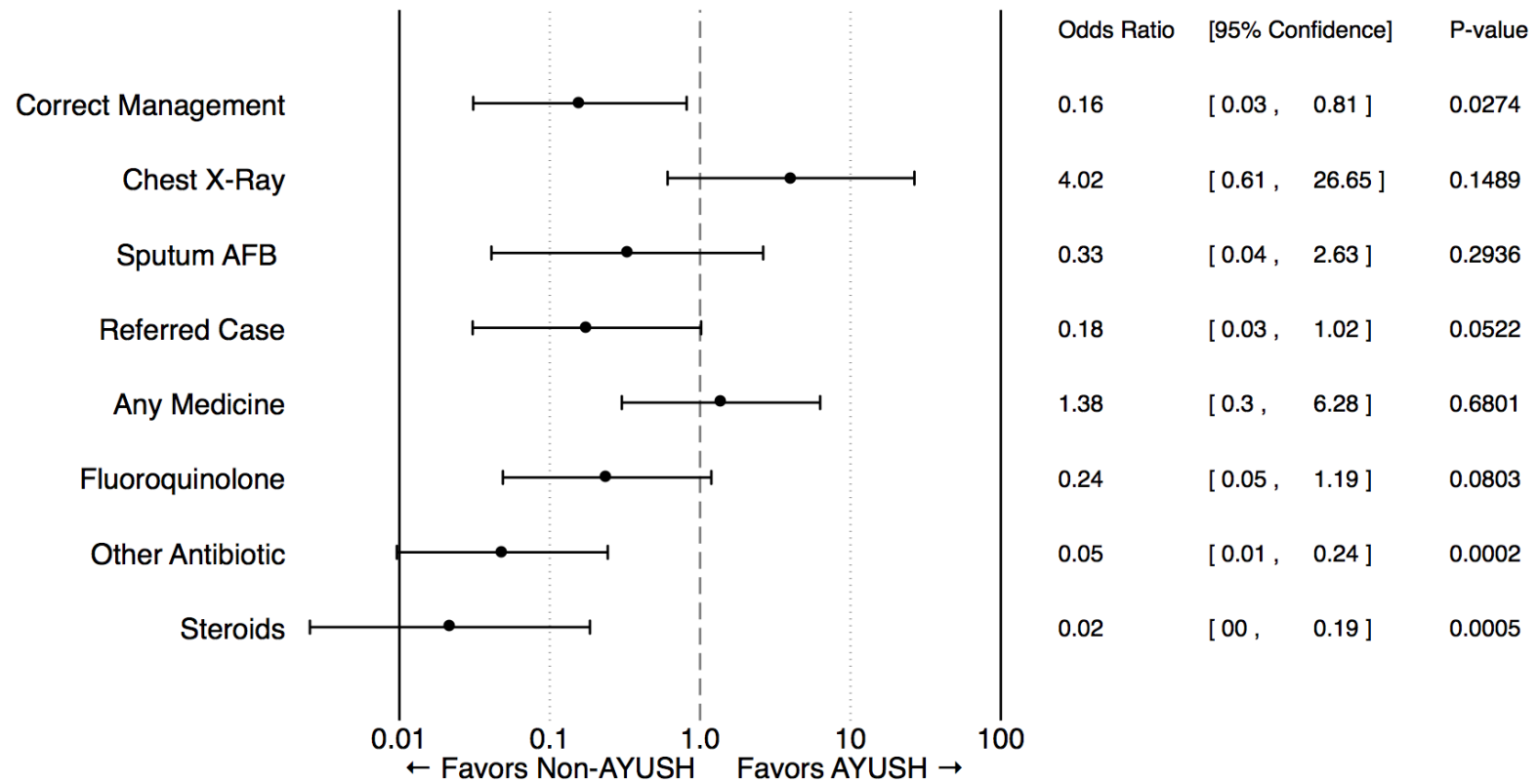

Supplement: S6 Fig — AYUSH, Ayurveda, Yoga, Unani, Siddha, or Homeopathy. (PDF) [file pmed.1002653.s010.pdf]
