# Supplementary material for: Variations in the quality of tuberculosis care in urban India: A cross-sectional, standardized patient study in two cities
Source: PLoS Med. 2018 Sep 25;15(9):e1002653. doi: 10.1371/journal.pmed.1002653 (PMC6155454; doi:10.1371/journal.pmed.1002653)

S2 Fig: Followup requests from providers by case management outcome, with alternate definitions

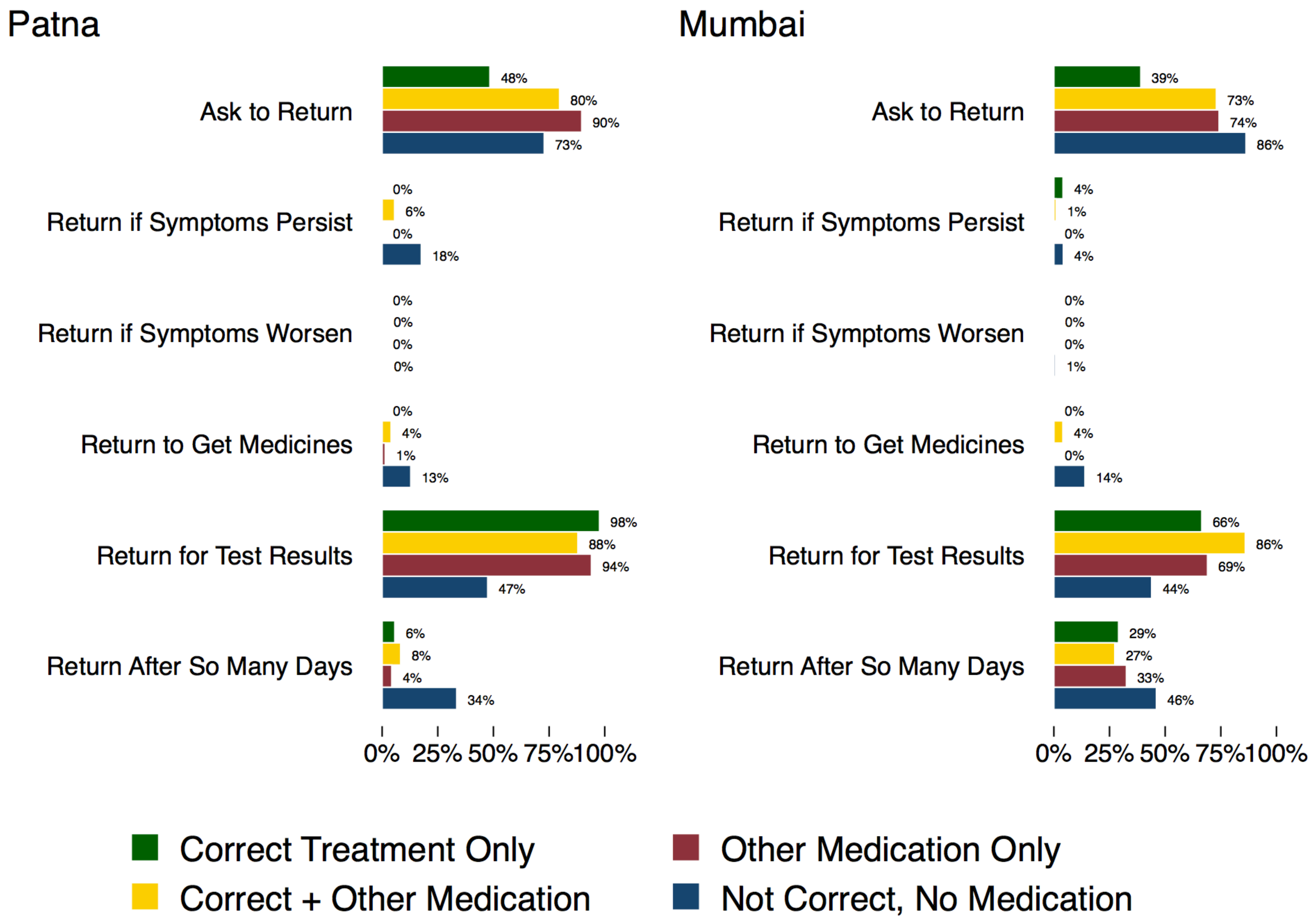

Supplement: S2 Fig — (PDF) [file pmed.1002653.s006.pdf]
