# Supplementary material for: Variations in the quality of tuberculosis care in urban India: A cross-sectional, standardized patient study in two cities
Source: PLoS Med. 2018 Sep 25;15(9):e1002653. doi: 10.1371/journal.pmed.1002653 (PMC6155454; doi:10.1371/journal.pmed.1002653)
Supplement: S7 Fig — (PDF) [file pmed.1002653.s011.pdf]

S7 Fig: Sampling flow diagram by primary strata

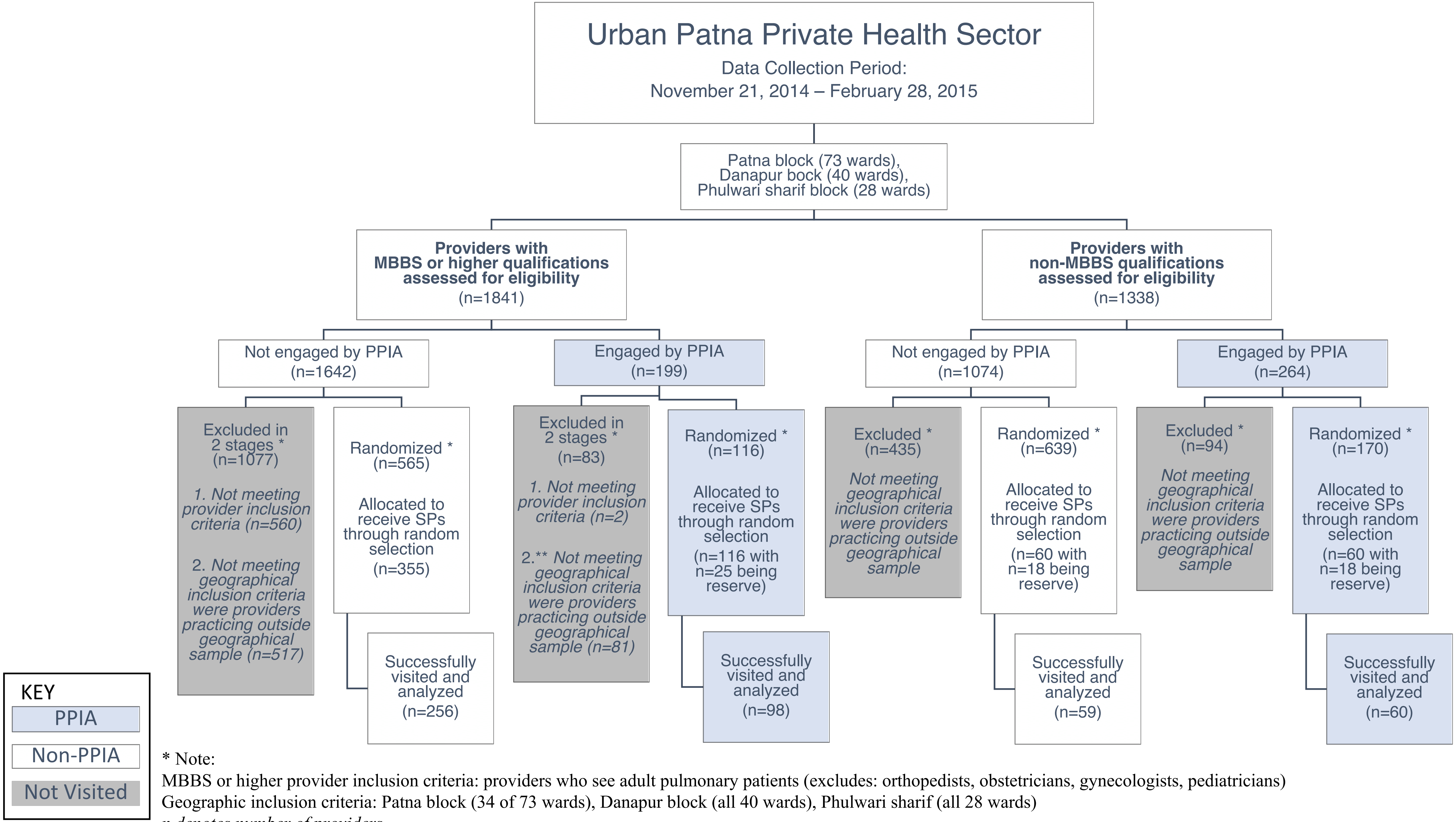

* Note:

MBBS or higher provider inclusion criteria: providers who see adult pulmonary patients (excludes: orthopedists, obstetricians, gynecologists, pediatricians)

Geographic inclusion criteria: Patna block (34 of 73 wards), Danapur block (all 40 wards), Phulwari sharif (all 28 wards)

n denotes number of providers

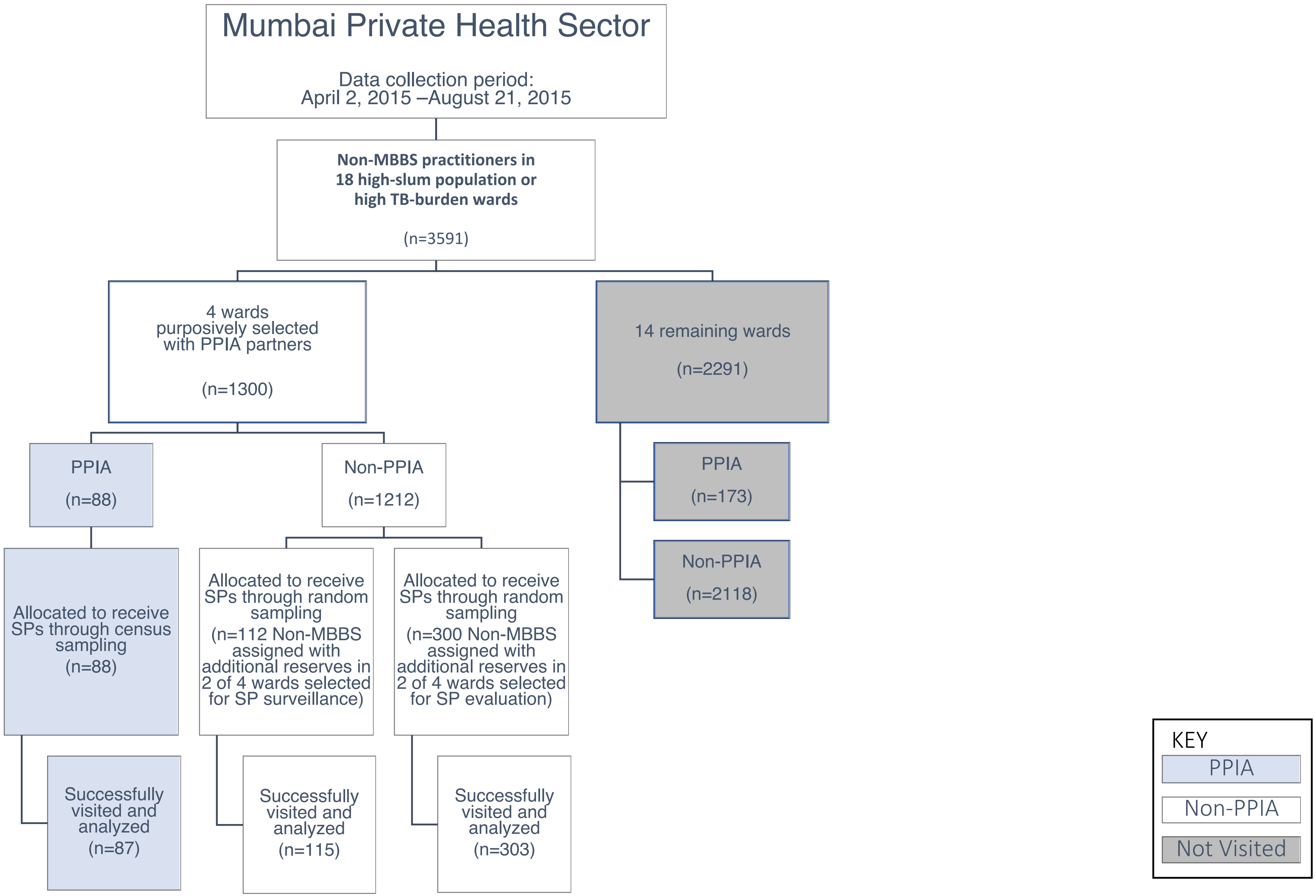
Mumbai Private Health Sector
Data collection period:
April 2, 2015 –August 21, 2015
Non-MBBS practitioners in 18 high-slum population or high TB-burden wards
(n=3591)
4 wards purposively selected with PPIA partners
(n=1300)
14 remaining wards
(n=2291)
PPIA
(n=88)
Non-PPIA
(n=1212)
PPIA
(n=173)
Non-PPIA
(n=2118)
Allocated to receive SPs through census sampling
(n=88)
Allocated to receive SPs through random sampling
(n=112 Non-MBBS assigned with additional reserves in 2 of 4 wards selected for SP surveillance)
Allocated to receive SPs through random sampling
(n=300 Non-MBBS assigned with additional reserves in 2 of 4 wards selected for SP evaluation)
Successfully visited and analyzed
(n=87)
Successfully visited and analyzed
(n=115)
Successfully visited and analyzed
(n=303)
KEY
PPIA
Non-PPIA
Not Visited

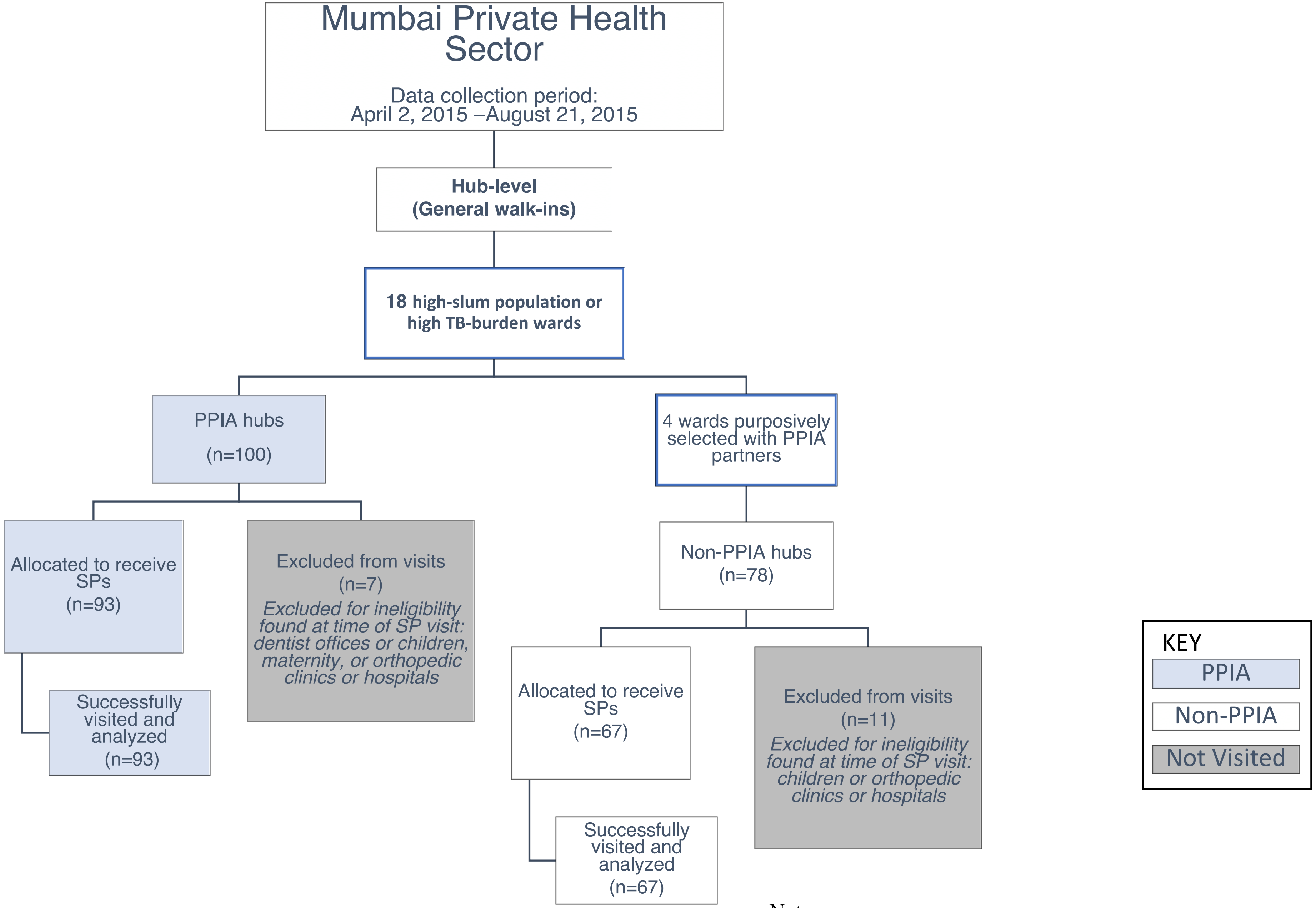

Note:

n denotes number of private health facilities (overlaps with PPIA provider interactions)

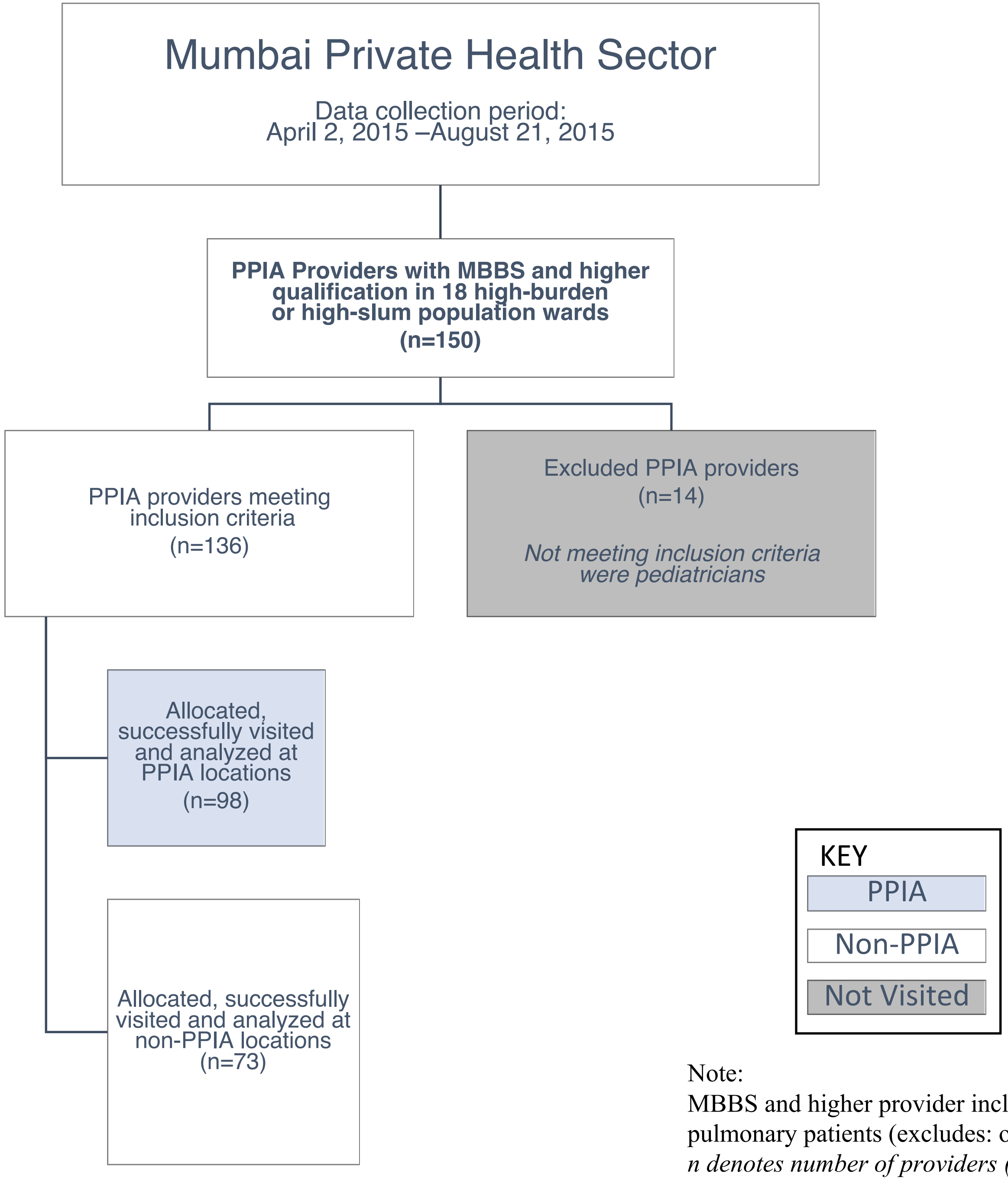

Note:
MBBS and higher provider inclusion criteria: providers who see adult pulmonary patients (excludes: orthopedists, pediatricians)
n denotes number of providers (overlaps with hub-level interactions)
