# Supplementary material for: Variations in the quality of tuberculosis care in urban India: A cross-sectional, standardized patient study in two cities
Source: PLoS Med. 2018 Sep 25;15(9):e1002653. doi: 10.1371/journal.pmed.1002653 (PMC6155454; doi:10.1371/journal.pmed.1002653)
Supplement: S1 Checklist — (PDF) [file pmed.1002653.s012.pdf]

STROBE Statement—checklist of items that should be included in reports of observational studies

	Item	Recommendation	Implementation
**Title and abstract**	1	(*a*) Indicate the study's design with a commonly used term in the title or the abstract	The title is "Variations in the quality of tuberculosis care in urban India: A cross-sectional, standardized patient study in two cities"
		(*b*) Provide in the abstract an informative and balanced summary of what was done and what was found	The abstract includes: "Between November 2014 and August 2015, unannounced adult standardized patients (SPs) portraying four TB "case scenarios" visited sampled health providers in Mumbai and Patna. Drawn from provider censuses, eligible providers were then selected randomly, stratified by the following provider qualification categories: (1) providers with MBBS (Bachelor of Medicine, Bachelor of Surgery) or higher degrees, and (2) non-MBBS providers, including licensed practitioners of alternative medicines and providers with other or no qualifications. The sampled 473 Patna providers represent 3,179 eligible providers and the sampled 730 Mumbai providers represent 7,116 eligible providers. Each provider received up to 4 SP visits at a given location."<br><br>The abstract continues to describe the four TB case scenarios, the two micro-experiments that were conducted, as well as the analysis.
**Introduction**			
Background / rationale	2	Explain the scientific background and rationale for the investigation being reported	In India, studies have demonstrated that the private health sector provides the bulk of primary care, is the first point of contact for 50-70% of patients with TB symptoms [3-5], and sells nearly twice the amount of anti-TB drugs when compared to the public sector [6]. However, navigating the private health sector can be clinically and economically catastrophic: TB patients who seek care experience a median of 33.5 days of diagnostic and treatment, convoluted pathways with multiple visits to an average of 2.7 providers before diagnosis, and broken continuum of care if diagnosed [4, 5, 7, 8]. With the emergence of drug resistant strains, these deficits may perpetuate disease transmission, hindering control efforts, particularly for high-density urban areas [9].<br><br>The Government of India now recognizes that engaging the private sector is critical to TB control and it has included this as an explicit goal in the Government's National Strategic Plan (NSP) for TB elimination (2017-2025). The NSP articulates a commitment to massively expand private provider engagement and calls for a six-fold increase in TB case notifications from the private sector, to two million patients per year by 2020 [11].

Objectives	3	State specific objectives, including any prespecified hypotheses	In order to provide rigorous measures on what explains levels of quality, our specific objective of this study is to report data on 2602 SP-provider interactions across 1203 representatively sampled health facilities in two cities. We additionally report results from two micro-experiments aimed to explain variation in our main outcome of interest correct case management.
**Methods**			
Study design	4	Present key elements of study design early in the paper	Study design section in Methods, "The standardized patient (SP) survey methodology consists of three steps: Creating a sampling frame, measuring quality, and analyzing the resulting data. We briefly describe each step here, with details in our supplemental appendices (S1-S3 Text)."
Setting	5	Describe the setting, locations, and relevant dates, including periods of recruitment, exposure, follow-up, and data collection	In the Methods section, "We conducted this study in the two Indian cities of Mumbai and Patna, where Private Provider Interface Agencies (PPIAs) were in the first year of implementing pilot, urban TB programs in the private sector [21, 22]. Patna is the capital of the state of Bihar, one of the least developed Indian states, with an annual per capita income of 30,000 Indian rupees (INR) (USD$470) and 1.7 million city inhabitants. Mumbai is the relatively wealthier, port capital of the state of Maharashtra, and is home to 12 million inhabitants with an annual per capita income of 180,000 INR (USD$2,845)."
Participants	6	*Cross-sectional study*—Give the eligibility criteria, and the sources and methods of selection of participants	See Provider and Facility Sampling in Methods section, "In both Mumbai and Patna, streetwise mapping exercises were conducted in 2014 to construct a comprehensive list of all providers and facilities in the private health sector. Providers eligible for the SP study were restricted to those known to see adult outpatients with respiratory symptoms in the private health sector; these include most primary care providers, but would exclude, for instance, gynecologists or ophthalmologists. Using the universe list, we then representatively sampled eligible providers from purposively sampled geographical areas within each city, which were identified for the SP study in the context of the PPIA pilot programs. Providers were selected with random sampling stratified by PPIA program enrollment status and provider qualification (Table 1). This was done to estimate baseline measures for quality of TB care in these cities and, in ongoing work (not published here), evaluate the impact of the programs (see S1 Text). In Mumbai, we sampled 225 MBBS facilities and 500 non-MBBS providers, and in Patna we sampled 474 MBBS and 120 non-MBBS providers. To estimate baseline quality measures, the data were weighted according to appropriate sampling proportions in both cities (S2 Text).SP cases were then assigned to providers to ensure that: (a) providers of all types would receive a mix of multiple SP case scenarios and (b) the risk of detection would be minimized. Interaction completion rates were uniformly high, with only Patna non-MBBS, non-PPIA providers having less than 85% of initially scheduled providers successfully visited (this proportion was 71%, due to many being discovered as specialized practitioners who did not treat respiratory conditions, such as cardiologists). "

Variables	7	Clearly define all outcomes, exposures, predictors, potential confounders, and effect modifiers. Give diagnostic criteria, if applicable	In Methods section, "Four distinct SP cases were developed and validated in a pilot study [12], our main outcome correct management was benchmarked against the Standards for TB Care in India (STCI) and the International Standards for TB Care (ISTC) [27, 28] (Table 2)."
Data sources/ measurement	8*	For each variable of interest, give sources of data and details of methods of assessment (measurement). Describe comparability of assessment methods if there is more than one group	*Four distinct SP cases were developed and validated in a pilot study [12], our main outcome correct management was benchmarked against the Standards for TB Care in India (STCI) and the International Standards for TB Care (ISTC) [27, 28] (Table 2). Each case was developed with a standardized opening statement and scripted presentation that would advance the provider towards a TB diagnosis and an appropriate case management action, which could include referral, laboratory testing, or treatment initiation, depending on the case scenario. Cases 1, 2, and 3 represented pulmonary TB at various stages of diagnostic certitude. Case 1 presents as a classic case of pulmonary TB with 2-3 weeks of cough and fever. Case 2 is similar to Case 1; however, the SP additionally has completed a one-week course of broad-spectrum antibiotics without any improvement and carries an abnormal chest X-ray (CXR) dated within 2 weeks of the interaction. Case 3 has visited the local government hospital and carries the results of a sputum smear microscopy acid-fast bacillus (AFB) test, which strongly suggests active TB. In all scenarios where the SP carried medical reports or images, the SPs conveyed to the provider that they did not know or understand what the reports showed—thereby varying the information available to the provider without altering the patient's revealed beliefs or expectations. 109 of the Case 1 interactions in Patna were completed at providers who had already completed a Case 1 interaction with a different SP actor, to assess the extent to which provider behavior is consistent across patients with identical presentations.Case 4 presents as a multi-drug resistant (MDR) TB suspect, with four weeks of cough and fever. The SP recalls receiving treatment from the government hospital in the past year for a similar condition and, if questioned, admits to a previous TB diagnosis with incomplete treatment adherence. All flags point towards TB recurrence, which should raise concern about drug-resistance. Among 50 of the sampled Mumbai MBBS or higher providers, we randomly assigned SPs to present an experimental variant of Case 4, who carried the same TB-positive sputum AFB report as in Case 3. With this variant, we aimed to analyze the effect of a TB-positive diagnostic test signal by comparing quality of care provided to SPs portraying Case 4 with vs. without the AFB report.*

Bias	9	Describe any efforts to address potential sources of bias	In introduction: "The SP method, which is considered the gold standard method to assess provider practice, has been increasingly used to capture levels of quality of care for TB and other health conditions [16-20]."<br><br>In limitations: "There are several limitations in our study. First, as we do not observe how patients actually choose providers, patient sorting by qualification, geography, personal relationships, price, reputation, or other unobserved signals of quality, prevents extrapolation to the likely outcomes for actual TB patients. This remains an important area for future work. Second, our cases are designed as one-time interactions, and the SP data do not reflect follow-up visit pathways, which have been shown by other studies to be long and convoluted and had various forms in our data (S2 Figure). For instance, we cannot say, from this study, what the doctor would do after the patient has returned after completing a CXR as ordered. To the extent that doctor behavior is different when the patient comes with a CXR that the doctor herself recommended (rather than with a CXR ordered by another doctor), our approximations of provider behavior under different scenarios may be erroneous."
Study size	10	Explain how the study size was arrived at	In study design (methods) section: "The standardized patient (SP) survey methodology consists of three steps: Creating a sampling frame, measuring quality, and analyzing the resulting data. We briefly describe each step here, with details in our supplemental appendices (S1-S3 Text)."
Quantitative variables	11	Explain how quantitative variables were handled in the analyses. If applicable, describe which groupings were chosen and why	We report raw proportions for outcomes of interest, with population mean estimates and confidence intervals computed using inverse probability weights based on the universe of private sector providers listed from the lane-by-lane mapping exercise in both cities (S1 Text, S2 Text). These weights are calculated such that each of the eight city-case combinations contribute equally to overall estimates. Within each city-case combination, individual interactions are weighted based on the actual proportion of providers enrolled and not enrolled in the PPIA in that city's provider sampling list within both the MBBS and non-MBBS strata versus the realized sample.<br><br>Therefore, the percentages reported for case management behaviors represent the estimated likelihood of the outcome occurring if a provider were chosen at random from the citywide population of providers, rather than the percentage of interactions in our sample in which the behavior was observed. In addition to using these weights to estimate population likelihoods, we use them to calculate odds ratios in logistic regressions comparing variation in quality of care across provider types, city settings, and SP case scenarios.

Statistical methods	12	(*a*) Describe all statistical methods, including those used to control for confounding	We report raw proportions for outcomes of interest, with population mean estimates and confidence intervals computed using inverse probability weights based on the universe of private sector providers listed from the lane-by-lane mapping exercise in both cities (S1 Text, S2 Text). These weights are calculated such that each of the eight city-case combinations contribute equally to overall estimates. Within each city-case combination, individual interactions are weighted based on the actual proportion of providers enrolled and not enrolled in the PPIA in that city's provider sampling list within both the MBBS and non-MBBS strata versus the realized sample. Therefore, the percentages reported for case management behaviors represent the estimated likelihood of the outcome occurring if a provider were chosen at random from the citywide population of providers, rather than the percentage of interactions in our sample in which the behavior was observed. In addition to using these weights to estimate population likelihoods, we use them to calculate odds ratios in logistic regressions comparing variation in quality of care across provider types, city settings, and SP case scenarios. In our supplementary results (S3 Text), we conduct ANOVA analysis to determine how well our primary stratification characteristics explain variations in SP management. Among the provider sample who received repeat Case 1 visits in Patna, we assess the level of consistency that those providers displayed across identical SP Case 1 visits using a different SP actor. We then use a quality proxy – checklist of history questions – to illustrate the amount of variation in provider behavior within each city-case combination. All analyses and programs were written in Stata 14 (College Station, TX).
		(*b*) Describe any methods used to examine subgroups and interactions	Examination of quality of care wsa done by qualification and setting: "One potential explanation is that the wide variation in management reflects systematic practice variation by qualification and/or setting, and we turn to this hypothesis next. Figure 3 reports odds ratios for differences in quality of care outcomes by qualification (top panel) and by city, stratified by qualification (middle and bottom panels). MBBS providers, who make up 58% of all providers in the Patna sampling list and 50% of all providers in the Mumbai list, were more likely than non-MBBS providers to correctly manage cases (OR 2.80; 95% CI 2.05–3.82), ask for CXR and/or sputum tests, and initiate anti-TB treatment. Despite providing relatively higher quality care, MBBS providers only correctly managed 709 of 1304 interactions (54%; 95% CI: 52–57). MBBS providers were also more likely than others to prescribe unnecessary or harmful antibiotics including FQs, although their steroid use was notably lower."
		(*c*) Explain how missing data were addressed	*N/A*

		(*d*) *Cohort study*—If applicable, explain how loss to follow-up was addressed	*N/A*
		Case-control study—If applicable, explain how matching of cases and controls was addressed	*N/A*
		Cross-sectional study—If applicable, describe analytical methods taking account of sampling strategy	*We report raw proportions for outcomes of interest, with population mean estimates and confidence intervals computed using inverse probability weights based on the universe of private sector providers listed from the lane-by-lane mapping exercise in both cities (S1 Text, S2 Text). These weights are calculated such that each of the eight city-case combinations contribute equally to overall estimates. Within each city-case combination, individual interactions are weighted based on the actual proportion of providers enrolled and not enrolled in the PPIA in that city's provider sampling list within both the MBBS and non-MBBS strata versus the realized sample.*
		(*e*) Describe any sensitivity analyses	N/A
**Results**			
Participants	13*	(a) Report numbers of individuals at each stage of study—eg numbers potentially eligible, examined for eligibility, confirmed eligible, included in the study, completing follow-up, and analysed	See Table 1, Supplemental Text S1d, and Supplemental Figure S7
		(b) Give reasons for non-participation at each stage	See Table 1, Supplemental Text S1d, and Supplemental Figure S7
		(c) Consider use of a flow diagram	See Supplemental Figure S7
Descriptive data	14*	(a) Give characteristics of study participants (eg demographic, clinical, social) and information on exposures and potential confounders	See Table 1 and Results section, "In our study, 1288 providers were successfully visited by SPs across both cities. Among these providers, the majority were male (88%), had a clinic assistant (65%), and fell into the age category of 30-50 years (71%), which were all characteristics observed by SPs during the interactions."
		(b) Indicate number of participants with missing data for each variable of interest	See Table 1, Supplemental Text S1d, and Supplemental Figure S7
		(c) *Cohort study*—Summarise follow-up time (eg, average and total amount)	*N/A*
Outcome data	15*	*Cohort study*—Report numbers of outcome events or summary measures over time	*N/A*
		Case-control study—Report numbers in each exposure category, or summary measures of exposure	*N/A*

		Cross-sectional study—Report numbers of outcome events or summary measures	*Figure 1 illustrates our main city-level estimates of average case management outcomes among the city-representative sample, with proportions estimated with weights to represent the estimated likelihood of the outcome occurring if a provider were chosen at random from the citywide population of providers.*
Main results	16	(*a*) Give unadjusted estimates and, if applicable, confounder-adjusted estimates and their precision (eg, 95% confidence interval). Make clear which confounders were adjusted for and why they were included	N/A
		(*b*) Report category boundaries when continuous variables were categorized	N/A
		(*c*) If relevant, consider translating estimates of relative risk into absolute risk for a meaningful time period	N/A
Other analyses	17	Report other analyses done—eg analyses of subgroups and interactions, and sensitivity analyses	In our supplementary results appendix, an ANOVA decomposition (S3 Figure) shows that our primary stratification characteristics of qualification and setting typically predicted less than 25% of the observed variation in each of our primary case management outcomes. By contrast, in a sub-sample where 109 Patna providers received a repeat Case 1 interaction (S4 Figure), consistency levels between the two visits were near 75% for all behaviors. Therefore, we observe that practice is highly variable across providers, even within the same setting and qualification strata, and therefore rule out these variables as explanatory of the wide variety in management practices.<br><br>Using essential history checklist completion as a proxy for the distribution of individualized quality levels (S5 Figure), our results suggest that there is a substantial and unexplained idiosyncratic component to quality that varies widely even within each city and qualification level. Rather than tight clustering around the group means, in all cases we observe a "long tail" of both low- and high-quality MBBS and non-MBBS providers in each city. Thus, moving beyond averages to full distributions of quality yields important and nuanced additional results for the estimation of "average" outcome quality, both by city and by qualification.
**Discussion**			

Key results	18	Summarise key results with reference to study objectives	TB is a persistent health challenge for India and is one of the top five causes of death between the age of 30 to 69 [31]. With India's goal of eliminating TB by 2025 as stated in the NSP, the success of this plan heavily depends on whether India's large, unregulated, and diverse private sector can be effectively engaged to identify missing TB patients and ensure that all TB patients receive sufficiently high levels of quality of care [32]. Our city-representative data from an at-scale study show significant deficits in the average provider's management of TB cases in both study cities. This low quality is characterized by underuse of appropriate diagnostics and widespread use of unnecessary medications, including antibiotics and contraindicated FQs. Even though MBBS-qualified providers degrees managed the SP cases better on average, there was still considerable variation within qualification in each setting and relatively little difference between the two cities on average.
Limitations	19	Discuss limitations of the study, taking into account sources of potential bias or imprecision. Discuss both direction and magnitude of any potential bias	First, as we do not observe how patients actually choose providers, patient sorting by qualification, geography, personal relationships, price, reputation, or other unobserved signals of quality, prevents extrapolation to the likely outcomes for actual patients with TB. This remains an important area for future work. Second, our cases are designed as one-time interactions, and the SP data do not reflect follow-up visit pathways, which have been shown by other studies to be long and convoluted and had various forms in our data (S2 Figure). For instance, we cannot say, from this study, what the doctor would do after the patient has returned after completing a CXR as ordered. To the extent that doctor behavior is different when the patient comes with a CXR that the doctor herself recommended (rather than with a CXR ordered by another doctor), our approximations of provider behavior under different scenarios may be erroneous. The ability for the SP method to measure quality of care measures in follow-up visits with the same SP individual in similar settings has not been published to our knowledge, and given the frequency of providers asking for patients to return exhibited in this study, it could be worthwhile to explore the potential for the method to assess whether the likelihood of receiving better care increases when a patient returns. With this, it would be important to understand the extent to which real patients return upon provider's request, as well as the necessary work needed so that SPs are not detected.

Interpretation	20	Give a cautious overall interpretation of results considering objectives, limitations, multiplicity of analyses, results from similar studies, and other relevant evidence	Our study has several strengths. First, we representatively sampled large numbers of private health providers in two Indian cities, and after weighting to the city universe of providers, we provide precise estimates of provider behavior at the city level. Second, by using unannounced SPs, we captured actual provider behavior, as compared to self-reported knowledge or practices. Given our prior work showing a big 'know-do gap' (the gap between what providers know and what they do in actual practice) [12], the SP methodology better reflects reality that any other method that can be used to measure quality of care. Third, our study included MBBS-qualified providers, as well as AYUSH providers and those with other or no qualifications, capturing the complexity of training within the Indian private healthcare sector. Fourth, by developing 4 different SP case presentations, we study how providers dealt with various stages of TB disease and varying levels of diagnostic certainty. Lastly, by assessing outcomes by city, provider qualification and type of case, we assess the most important sources of variation in quality of care. While previous studies showed suboptimal quality of care, our study is the first to explore provider indicators of quality with city-representative samples of providers.
Generalisability	21	Discuss the generalisability (external validity) of the study results	Our study has several strengths and contributes to the literature in several ways. First, we representatively sampled large numbers of private health providers in two Indian cities, and after weighting to the city universe of providers, we provide precise estimates of provider behavior at the city level. Because the analysis is representative to these two Indian cities, caution is warranted when generalizing to a context outside of urban Indian setting
**Other information**			
Funding	22	Give the source of funding and the role of the funders for the present study and, if applicable, for the original study on which the present article is based	In the acknowledgments section, "This study was funded by Grand Challenges Canada (grant S5 0373-01), the Bill & Melinda Gates Foundation (grant OPP1091843), and the Knowledge for Change Program at the World Bank."
